# Intermittent epidural bolus versus continuous epidural infusions for labor analgesia: A meta-analysis of randomized controlled trials

**DOI:** 10.1371/journal.pone.0234353

**Published:** 2020-06-12

**Authors:** Xian Liu, Huan Zhang, Haijing Zhang, Mengzhuo Guo, Yuanchao Gao, Chunyan Du

**Affiliations:** Department of Anesthesiology, Beijing Tsinghua Changgung Hospital, School of Clinical Medicine, Tsinghua University, Beijing, China; Cleveland Clinic, UNITED STATES

## Abstract

There are inconsistent results regarding the efficacy and safety of intermittent epidural bolus (IPB) versus continuous epidural infusions (CPI) for labor analgesia. This study used a meta-analytic approach to assess the safety and treatment efficacy of IPB versus CPI for labor analgesia based on randomized controlled trials (RCTs). Four electronic databases were used to identify eligible RCTs. Pooled effect estimates at 95% confidence intervals (CIs) were calculated using a random-effects model. Twenty-two RCTs with 2,573 parturients were selected for final analysis. The findings revealed no significant differences between IPB and CPI for the incidences of cesarean and instrumental delivery. IPB was shown to be associated with shorter total duration of labor [weighted mean difference (WMD): −21.46; 95% CI: −25.07 to −17.85; P < 0.001], duration of the first of stage of labor (WMD: −13.41; 95% CI: −21.01 to −5.81; P = 0.001), and duration of the second stage of labor (WMD: −4.98; 95% CI: −9.32 to −0.63; P = 0.025). Furthermore, IPB significantly reduced the incidences of required anesthetic interventions compared with CPI [relative risk (RR): 0.61; 95% CI: 0.39–0.95; P = 0.030], whereas there was no significant difference between IPB and CPI for the time required in the first anesthetic intervention (WMD: 7.73; 95% CI: −33.68–49.15; P = 0.714). The local anesthetic IPB (bupivacaine equivalents) was associated with lower milligrams per hour of local anesthetic (WMD: −0.89; 95% CI: −1.41 to −0.36; P = 0.001) and better maternal satisfaction (WMD: 8.76; 95% CI: 4.18–13.35; P < 0.001). There were no significant differences between IPB and CPI for the risk of adverse events. This study found that parturients with IPB have short total duration of labor and duration of the first and second stage of labor, reduced requirements for additional anesthetic interventions, and improved maternal satisfaction.

## Introduction

Childbirth is arguably one of the most painful experiences for parturients [1]. The degree of pain experienced and relief could affect maternal satisfaction during the birthing process, resulting in long-term emotional and psychological effects [2]. A study has recommended the need for an effective analgesic strategy for the improvement of maternal satisfaction with minimal adverse events [3]. Another study has demonstrated that the analgesic effects of labor neuraxial analgesia were superior to those of parenteral opioids, nitrous oxide, and other non-pharmacologic strategies, with limited effects on the mode of delivery and outcomes in mothers and neonates [4]. Moreover, patient-controlled epidural analgesia (PCEA) was associated with better maternal satisfaction and lower risk of adverse events, whereas the use of PCEA monotherapy did not yield a significant effect on pain control in parturients and clinician’s workload [5, 6].

At present, the continuous epidural infusions (CEI) technique is a standard labor epidural analgesic regimen in North America and Europe irrespective of PCEA status [7, 8]. However, the risk of instrumental delivery is high and the duration of the second stage of labor is prolonged in parturients receiving CEI [9]. A study has reported that regularly spaced intermittent boluses may result in more extensive spread of local anesthetic in the epidural space [10]. Therefore, the same dose of the local anesthetic given via intermittent epidural bolus (IPB) might improve analgesic effects. However, there are inconsistent results regarding the treatment efficacies of IPB and CEI for labor analgesia. Therefore, the current meta-analysis was conducted based on randomized controlled trials (RCTs) to determine the efficacy and safety of IPB and CPI for labor analgesia.

## Materials and methods

### Data sources, search strategy, and selection criteria

The Preferred Reporting Items for Systematic Reviews and Meta-Analyses (PRISMA) Statement was used as a guide for conducting this study [11]. The study was designed as an RCT that compared the efficacy and safety of IPB and CPI for the eligibility of labor analgesia. However, publication language and status were not restricted. The electronic databases of PubMed, EmBase, the Cochrane library, and Chinese National Knowledge Infrastructure were systematically searched for potentially included studies from their inception to November 2019 using “epidural” and/or “intermittent”/”programmed”/”automated” and “continuous” and “randomized controlled trials” as core search terms. The details of the search strategy in PubMed are displayed in S1 Appendix. The reference lists of the retrieved studies were also reviewed to identify whether the study met the inclusion criteria. The inclusion criteria of this study were as follows:

(1) Participants: Parturients;
(2) Intervention: IPB;
(3) Control: CPI;
(4) Outcomes: Cesarean delivery, instrumental delivery, total duration of labor, duration of the first stage of labor, duration of the second stage of labor, required anesthetic interventions, time to first required anesthetic intervention, milligrams per hour of local anesthetic (bupivacaine equivalents), maternal satisfaction, and potential adverse events;
(5) Study design: RCT.

### Data collection and quality assessment

The variables in each trial were abstracted, including first authors’ name; publication year; country; sample size; age; parity status; neuraxial analgesia initiation; epidural maintenance solution; IPE, CPI, or PCEA strategies; spontaneous/induced labor; and reported outcomes (S2 Appendix). The quality of the included studies was assessed using the Jadad scale based on randomization, blinding, allocation concealment, withdrawals and dropouts, and the use of intention-to-treat analysis [12]. The scoring system of the Jadad scale ranged from 0 to 5, with studies scoring 4 or 5 deemed as high quality.

### Statistical analysis

The efficacy and safety of IPB compared with CPI for labor analgesia were assigned as categorical and continuous data, and the relative risks (RRs) or weighted mean differences (WMDs) with 95% confidence intervals (CIs) were calculated before data pooling. The pooled analyses for all outcomes using the random-effects model were considered as the underlying variables across included trials [13, 14]. Heterogeneity among the included trials was assessed using *I*^*2*^ and Q-statistic with *I*^*2*^ > 50% or P < 0.10 regarded as significantly heterogeneous [15, 16]. The robustness of the pooled conclusion was assessed using sensitivity analysis by sequentially excluding individual trials using the random-effects model [13, 14, 17]. Moreover, subgroups analysis for the efficacy of outcomes was conducted based on PCEA status and differences between subgroups evaluated using interaction P-value, which assumes that the value in the subgroup meets the normal distribution and calculated *t*-test [18]. Publication biases were assessed using funnel plots and Egger and Begg test results [19, 20]. The inspection level for pooled results was two-sided, and P < 0.05 was considered as statistically significant. The STATA software (Version 10.0; StataCorp, Texas, United States of America) was used to conduct all analysis in this study.

## Results

### Literature search

**Fig 1:**
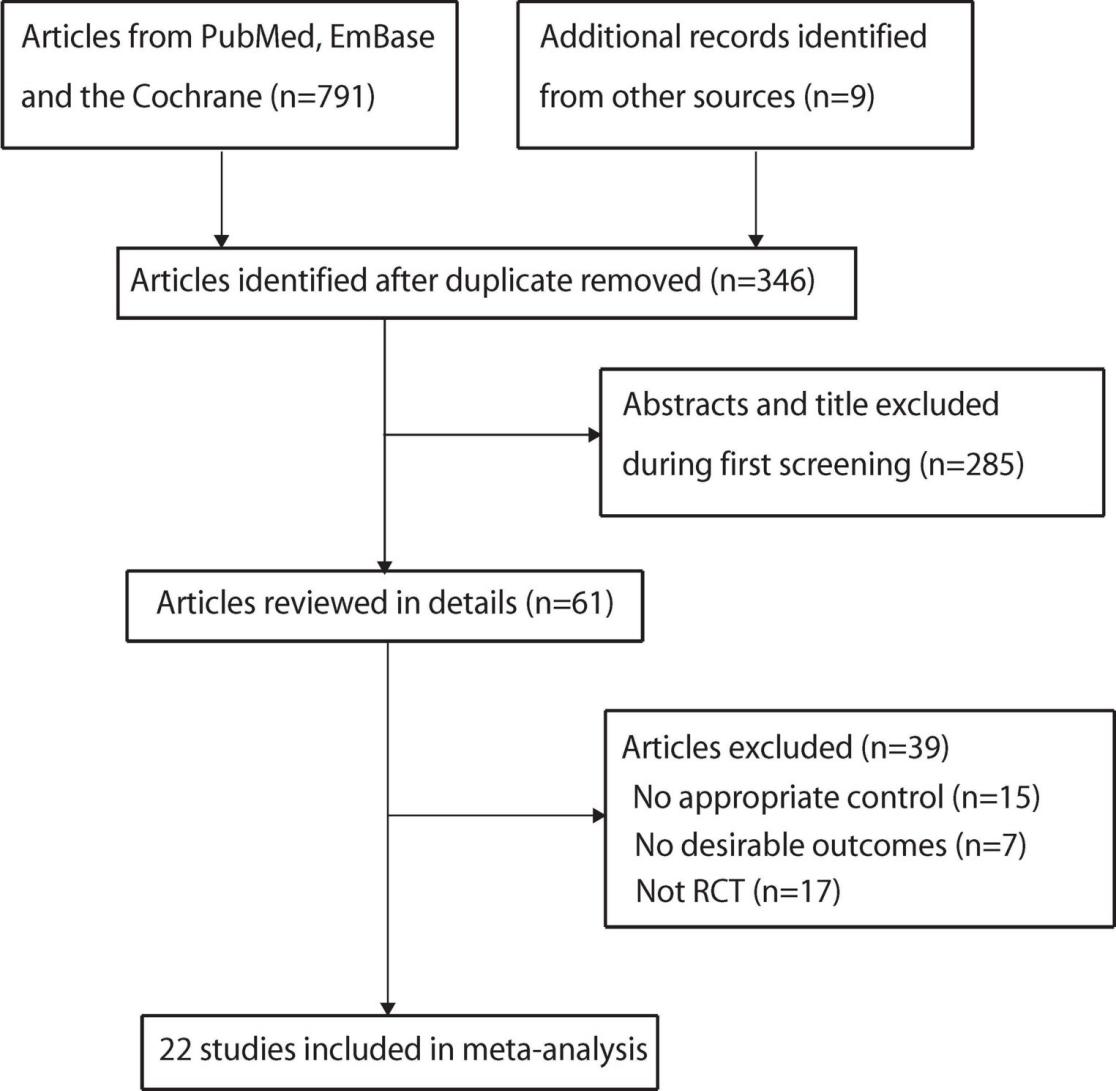
The PRISMA flowchart regarding the study selection process.

The PRISMA flowchart shows the details of the study selection process (Fig 1). The initial electronic database search yielded 791 records; 445 articles were excluded owing to duplicated topics. Another 285 studies were excluded owing to irrelevant topics after reviewing the title and abstracts. The remaining 61 studies were retrieved for full-text evaluations; 39 articles were excluded as they lacked appropriate control (15), desirable outcomes (7), and RCT design (17). The reviewed reference lists of the 22 RCTs yielded 9 potentially included studies. All these studies were present in electronic searches and were used for final quantitative analysis [21–42].

### Study characteristics

A total of 2,573 parturients from 22 RCTs were used. The baseline characteristics of the studies and participants are summarized in Table 1. The studies published between 2004 and 2019 included 40–205 parturients in each individual trial. Sixteen studies were conducted in Asia, while the remaining six studies were conducted in US or Europe. Eighteen studies included nulliparous parturients; two included parous parturients; and the remaining included both nulliparous and parous parturients. Sixteen studies included participants who received PCEA, while the remaining six studies included patients who did not receive PCEA. Study quality was evaluated using the Jadad scale: eight trials scored 5, five trials scored 4, and the remaining nine trials scored 3.

**Table 1:** The baseline characteristics of the included participants and studies.

Study	Country	Sample size	Age (years)	Parity	Neuraxial analgesia initiation	Epidural maintenance solution	IEP	CEI	PCEA	Spontaneous/induced labor	Study quality
Chua 2004 [21]	Singapore	42 (21/21)	NA	Nulliparous	EA (fentanyl, 25 µg)	Ropivacaine, 0.1%; fentanyl 2 µg/mL	5 mL bolus every hour	5 mL/h	No	Spontaneous labor; preanalgesia oxytocin; CEI, 8; IEB, 9	4
Lim 2005 [22]	Singapore	60 (30/30)	30.5	Nulliparous	EA (fentanyl, 25 µg)	Levobupivacaine, 0.1%; fentanyl, 2 µg/mL	5 mL bolus every 30 min	10 mL/h	No	Preanalgesia oxytocin; CEI, 4; IEB, 9	3
Salim 2005 [23]	Israel	127 (64/63)	25.6	Nulliparous	NA	Bupivacaine, 0.125%- 0.25%; fentanyl, 2 µg/mL	10 mL bolus every hour (0.25%)	8 mL/h (0.125%)	Yes (3 mL bolus, 20-min lockout)	Induction of labor; CEI, 17; IEB, 14	4
Fettes 2006 [24]	UK	40 (20/20)	26.5	Nulliparous	NA	Ropivacaine, 0.2%; fentanyl, 2 µg/mL	10 mL bolus every hour	10 mL/h	No	Induction of labor; CEI, 12; IEB, 14	5
Wong 2006 [25]	US	126 (63/63)	NA	Parous	SA (bupivacaine, 1.25 mg; fentanyl, 15 µg)	Bupivacaine, 0.625%; fentanyl, 2 µg/mL	6 mL bolus every 30 min	12 mL/h	Yes (5 mL bolus, 10-min lockout)	Induction of labor; CEI, 63; IEB; 63	5
Sia 2007 [26]	Singapore	42 (21/21)	NA	Nulliparous	SA (ropivacaine, 2 mg; fentanyl, 15 µg)	Ropivacaine 0.1%; fentanyl, 2 µg/mL	5 mL bolus every hour	5 mL/h	Yes (5 mL bolus, 10-min lockout)	Spontaneous labor; preanalgesia oxytocin; CEI, 5; IEB, 7	5
Lim 2010 [27]	Singapore	50 (25/25)	NA	Nulliparous	SA (ropivacaine, 2 mg; fentanyl, 15 µg)	Ropivacaine, 0.1%; fentanyl, 2 µg/mL	2.5 mL bolus every 15 min	10 mL/h	No	Spontaneous labor; Preanalgesia oxytocin; CEI, 10; IEB, 5	5
Leo 2010 [28]	Singapore	62 (31/31)	NA	Nulliparous	SA (ropivacaine, 2 mg; fentanyl, 15 µg)	Ropivacaine 0.1%; fentanyl, 2 µg/mL	5 mL bolus every hour	5 mL/h	Yes (5 mL bolus, 10-min lockout)	Preanalgesia oxytocin; CEI, 10; IEB, 15	4
Capogna 2011 [29]	Italy	145 (75/70)	28.0	Nulliparous	EA (levobupivacaine, 0.0625%; sufentanil, 10 µg/mL; 20 mL)	Levobupivacaine, 0.0625%–0.125%; sufentanil, 0.5 µg/mL	10 mL (0.0625%) bolus every hour	10 mL/h (0.0625%)	Yes (5 mL bolus, 10-min lockout, 0.125%)	Spontaneous labor; oxytocin use not reporte	5
Skrablin 2011 [30]	Croatia	205 (101/104)	28.0	Nulliparous	EA (levobupivacaine 20 ml, 0.07% with 2 mg/mL fentanyl)	Levobupivacaine 20 ml, 0.07% with 2.5 µg/mL fentanyl	20 mL bolus every hour	14 mL/h	No	Spontaneous labor; preanalgesia oxytocin; CEI, 50; IEB, 54	3
Sia 2013 [31]	Singapore	102 (51/51)	NA	Nulliparous	SA (ropivacaine, 2 mg; fentanyl, 15 µg)	Ropivacaine 0.1%; fentanyl, 2 µg/mL	5 mL (0.1%) bolus every hour	5 mL/h (0.1%)	Yes (5 mL bolus, 10-min lockout, 0.1%)	Preanalgesia oxytocin; CEI, 18; IEB, 14	5
Zhao 2013 [32]	China	57 (29/28)	25.0	Parous	SA (ropivacaine, 3 mg)	Ropivacaine 0.1%; sufentanil, 0.5 µg/mL	3 mL (0.1%) bolus every hour	6 mL/h (0.1%)	Yes (3 mL bolus, 10-min lockout, 0.1%)	Preanalgesia oxytocin; CEI, 6; IEB, 8	3
Feng 2014 [33]	China	125 (63/62)	27.5	Nulliparous	EA (10 ml of 0.125% ropivacaine; 0.4 µg/mL sufentanil)	0.08% ropivacaine; 0.4 µg/mL sufentanil	10 mL bolus every hour	10 mL/h	Yes (5 mL bolus, 30-min lockout)	Preanalgesia oxytocin; CEI, 44; IEB, 40	4
Lin 2016 [34]	China	200 (100/100)	27.8	Nulliparous	EA (ropivacaine, 0.15%; 10 mL)	Ropivacaine, 0.1%; sufentanil, 0.3 µg/mL	5 mL (0.1%) bolus every hour	5 mL/h (0.1%)	Yes (5 mL bolus, 20-min lockout, 0.1%)	Spontaneous labor and oxytocin use not reported	5
Nunes 2016 [35]	Portugal	130 (70/60)	29.0	Both	EA (10 mL of 0.16% ropivacaine plus sufentanil, 10 µg)	Ropivacaine 0.15%; sufentanil 0.2 µg/mL	10 mL bolus every hour	5 mL/h	Yes (5 mL bolus, 20-min lockout)	Spontaneous labor and oxytocin use not reported	4
Maggiore 2016 [36]	Italy	104 (52/52)	32.9	Both	EA (20 mL of 0.0625% levobupivacaine plus sufentanil, 10 µg)	0.0625% levobupivacaine with sufentanil 0.5 µg/mL	10 mL bolus every hour	10 mL/h	No	Spontaneous labor and oxytocin use not reported	5
Ji 2016 [37]	China	50 (25/25)	28.0	Nulliparous	EA (ropivacaine, 0.075%; sufentanil, 0.3 µg/mL; 8 mL)	Ropivacaine, 0.075%; sufentanil, 0.3 µg/mL	8 mL (0.075%) bolus every hour	8 mL/h (0.075%)	Yes (5 mL bolus, 20-min lockout, 0.075%)	Preanalgesia oxytocin; CEI, 21; IEB, 24	3
Fang 2016 [38]	China	200 (100/100)	24.6	Nulliparous	EA (ropivacaine, 0.075%; sufentanil, 0.5 µg/mL; 10 mL)	Ropivacaine, 0.075%; sufentanil, 0.5 µg/mL	8 mL (0.075%) bolus every hour	8 mL/h (0.075%)	Yes (6 mL bolus, 15-min lockout, 0.075%)	Spontaneous labor; preanalgesia oxytocin; CEI, 67; IEB, 62	3
Wang 2016 [39]	China	200 (100/100)	27.5	Nulliparous	EA (ropivacaine, 0.125%; sufentanil, 0.4 µg/mL 10 mL)	Ropivacaine 0.08%; sufentanil, 0.4 µg/mL	10 mL (0.08%) bolus every hour	10 mL/h (0.08%)	Yes (5 mL bolus, 30-min lockout, 0.1%)	Spontaneous labor and oxytocin use not reported	3
Wang 2017 [40]	China	186 (124/62)	26.0	Nulliparous	EA (ropivacaine, 0.125%; sufentanil, 0.4 µg/mL 10 mL)	Ropivacaine 0.08%; sufentanil, 0.4 µg/mL	10 mL (0.08%) bolus every hour	10 mL/h (0.08%)	Yes (5 mL bolus, 30-min lockout, 0.1%)	Spontaneous labor; preanalgesia oxytocin; CEI, 24; IEB, 36	3
Wang 2019 [41]	China	120 (60/60)	26.8	Nulliparous	EA (ropivacaine, 0.08%; sufentanil, 0.4 µg/mL 10 mL)	Ropivacaine, 0.08%; sufentanil, 0.4 µg/mL	10 mL (0.08%) bolus every hour	10 mL/h (0.08%)	Yes (5 mL bolus, 30-min lockout)	Spontaneous labor; preanalgesia oxytocin; CEI, 12; IEB, 4	3
Zhao 2019 [42]	China	200 (100/100)	NA	Nulliparous	EA (ropivacaine, 0.10%; sufentanil, 0.45 µg/mL)	Ropivacaine, 0.10%; sufentanil, 0.45 µg/mL	6 mL bolus every hour	6 mL/h	Yes (6 mL bolus, 15-min lockout)	Spontaneous labor and oxytocin use not reportes	3

### Cesarean and instrumental deliveries

**Fig 2:**
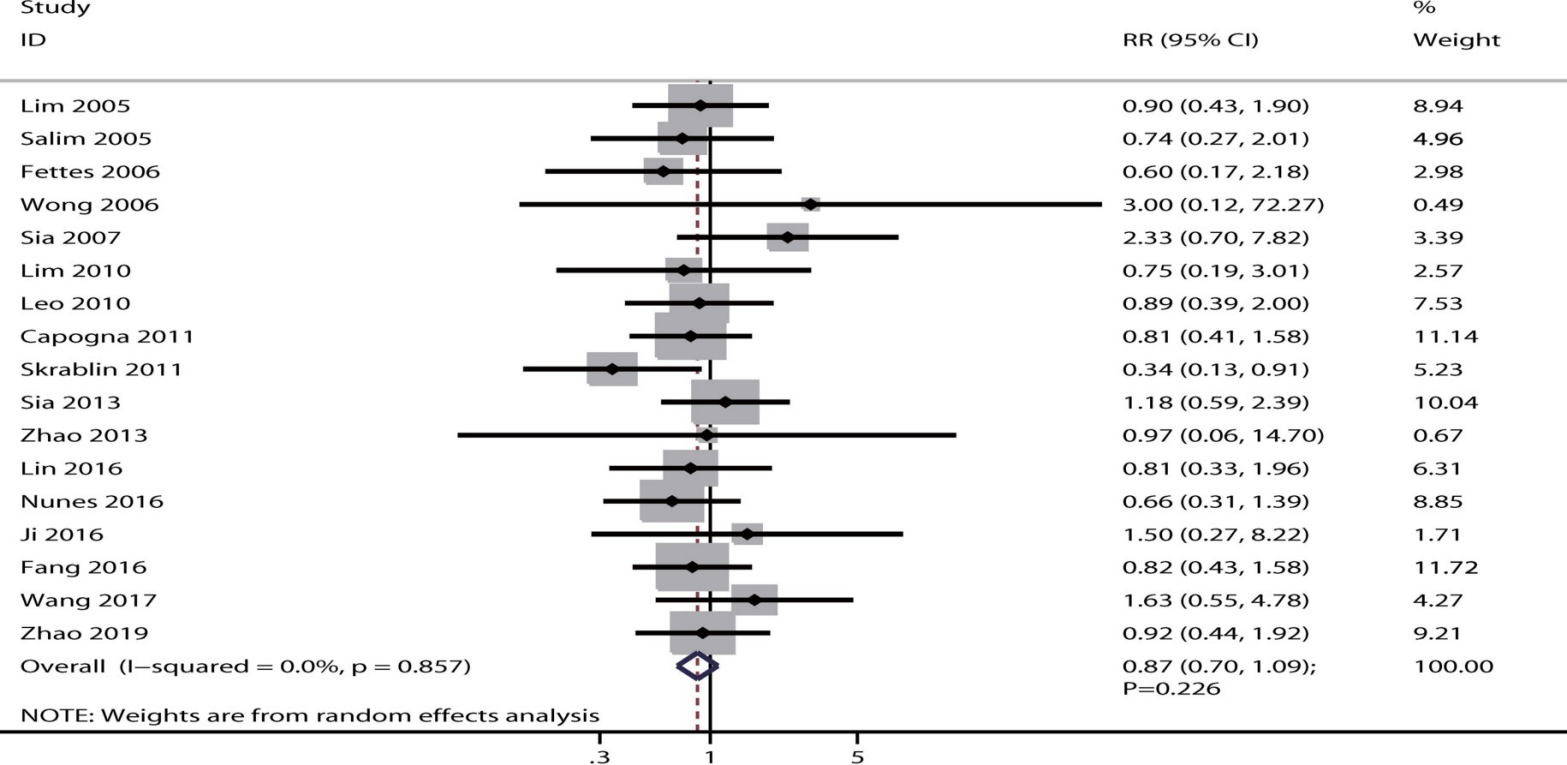
Effect of IPB versus CPI on the incidence of cesarean delivery.

The data for the effect of IPB versus CPI on the incidence of cesarean delivery were available in 17 studies. The pooled results indicated no significant difference between IPB and CPI for the incidence of cesarean delivery [RR: 0.87; 95% CI: 0.70–1.09; P = 0.226; no evidence of heterogeneity (*I*^*2*^ = 0.0%; P = 0.857), as shown in Fig 2]. The conclusion was robust and did not change by excluding any particular trial (S3 Appendix). The results of subgroups analysis indicated no statistical significance between IPB and CPI on cesarean delivery irrespective of PCEA status (Table 2). Although the symmetry for funnel plot could not rule out potential publication bias, the quantitative tests found no significant publication bias for cesarean delivery (P-values for Egger and Begg tests: 0.361 and 0.592, respectively; S4 Appendix).

**Table 2:** Subgroup analyses based on PCEA status.

Outcomes	Group	No. of studies	WMD or RR and 95% CI	P-value	*I*^*2*^ (%)	P-value for Q-statistic	P-value for subgroup effect
Cesarean	Yes	13	0.94 (0.73–1.21)	0.626	0.0	0.919	0.190
	No	4	0.64 (0.39–1.06)	0.081	0.0	0.472	
Instrumental	Yes	13	0.75 (0.47–1.20)	0.235	50.4	0.019	0.698
	No	3	0.89 (0.53–1.50)	0.660	0.0	0.609	
Total duration (minutes) of labor	Yes	13	−21.27 (−24.92–−17.62)	<0.001	0.0	0.583	0.511
	No	4	−50.57 (−97.81–−3.34)	0.036	37.0	0.190	
Duration of the first stage of labor	Yes	11	−12.80 (−20.45–−5.14)	0.001	53.5	0.018	0.227
	No	2	−61.31 (−128.58–5.96)	0.074	0.0	0.453	
Duration of the second stage of labor	Yes	10	−4.78 (−9.21–−0.35)	0.035	91.1	<0.001	0.729
	No	2	−13.72 (−40.84–13.41)	0.322	0.0	0.517	
Required anesthetic interventions	Yes	5	0.56 (0.34–0.91)	0.018	25.3	0.253	0.031
	No	4	0.64 (0.30–1.38)	0.253	76.7	0.005	
Time to first required anesthetic intervention	Yes	2	−15.99 (−47.77 to 15.79)	0.324	0.0	0.506	<0.001
	No	4	15.60 (−32.81–64.01)	0.528	81.0	0.001	
Milligrams per hour of local anesthetic (bupivacaine equivalents)	Yes	9	−1.12 (−1.74–−0.51)	<0.001	77.1	<0.001	0.012
	No	3	−0.11 (−0.85–0.63)	0.761	19.1	0.290	
Maternal satisfaction	Yes	9	9.25 (4.05–14.45)	<0.001	98.4	<0.001	0.001
	No	2	6.58 (3.64–9.51)	<0.001	11.0	0.289	

**Fig 3:**
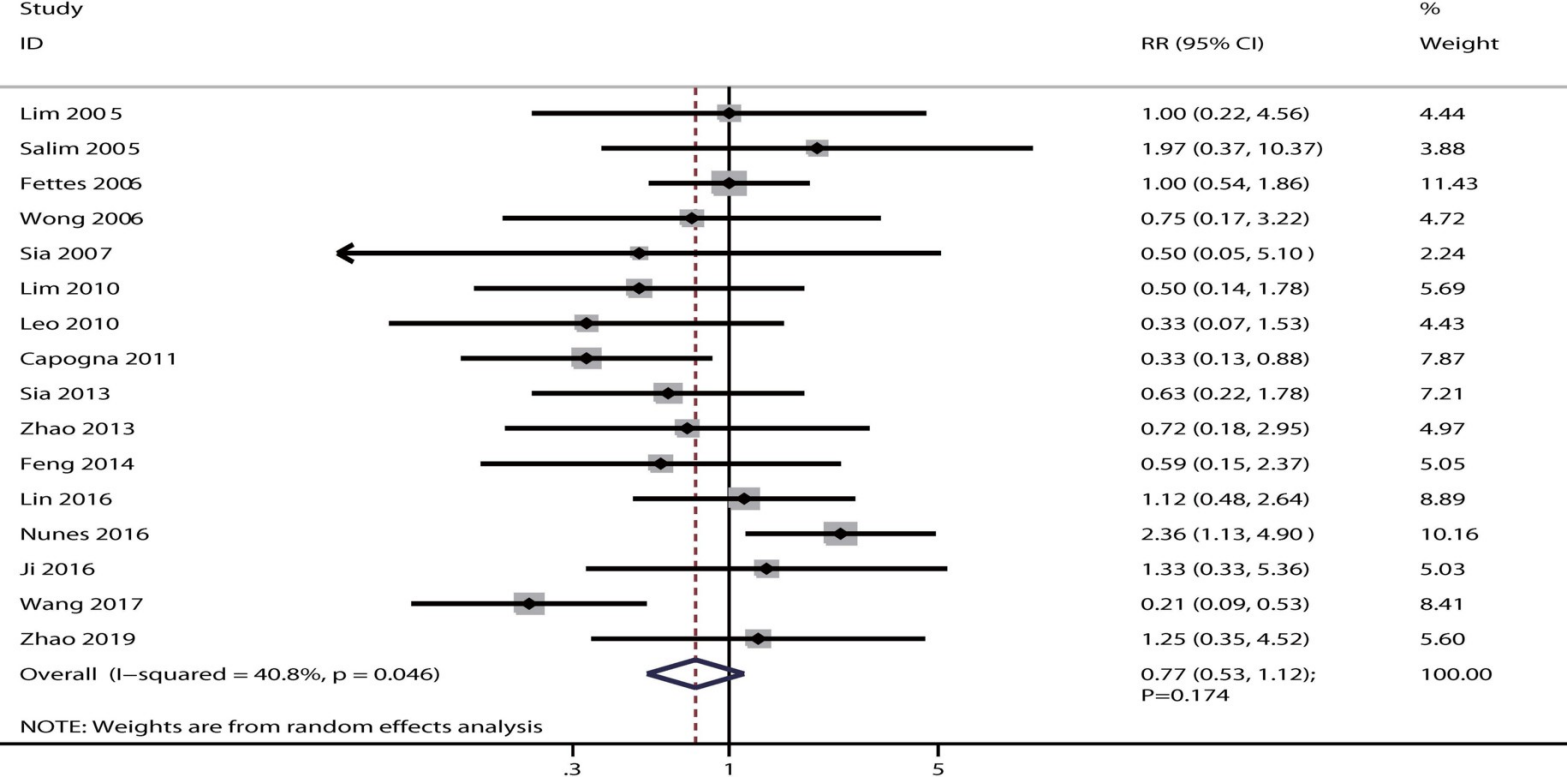
The effect of IPB versus CPI on the incidence of instrumental delivery.

The data for the effect of IPB versus CPI on the incidence of instrumental delivery were available in 16 studies. There was no significant difference between IPB and CPI on the incidence of instrumental delivery [RR: 0.77; 95% CI: 0.53–1.12; P = 0.174; potential significant heterogeneity (*I*^*2*^ = 40.8%; P = 0.046]; Fig 3). Sensitivity analysis indicated that IPB was associated with a lower incidence of instrumental delivery than CPI after excluding the study conducted by Nunes et al. [35], which specifically included both nulliparous and parous parturients (S3 Appendix). Subgroup analysis did not observe significant difference between IPB and CPI on instrumental delivery when stratified by PCEA status (Table 2). The symmetry for funnel plot for instrumental delivery was good, and no significant publication bias was detected for instrumental delivery when tested using the Egger (P = 0.433) and Begg (P = 1.000) tests (S4 Appendix).

### Duration of labor

**Fig 4:**
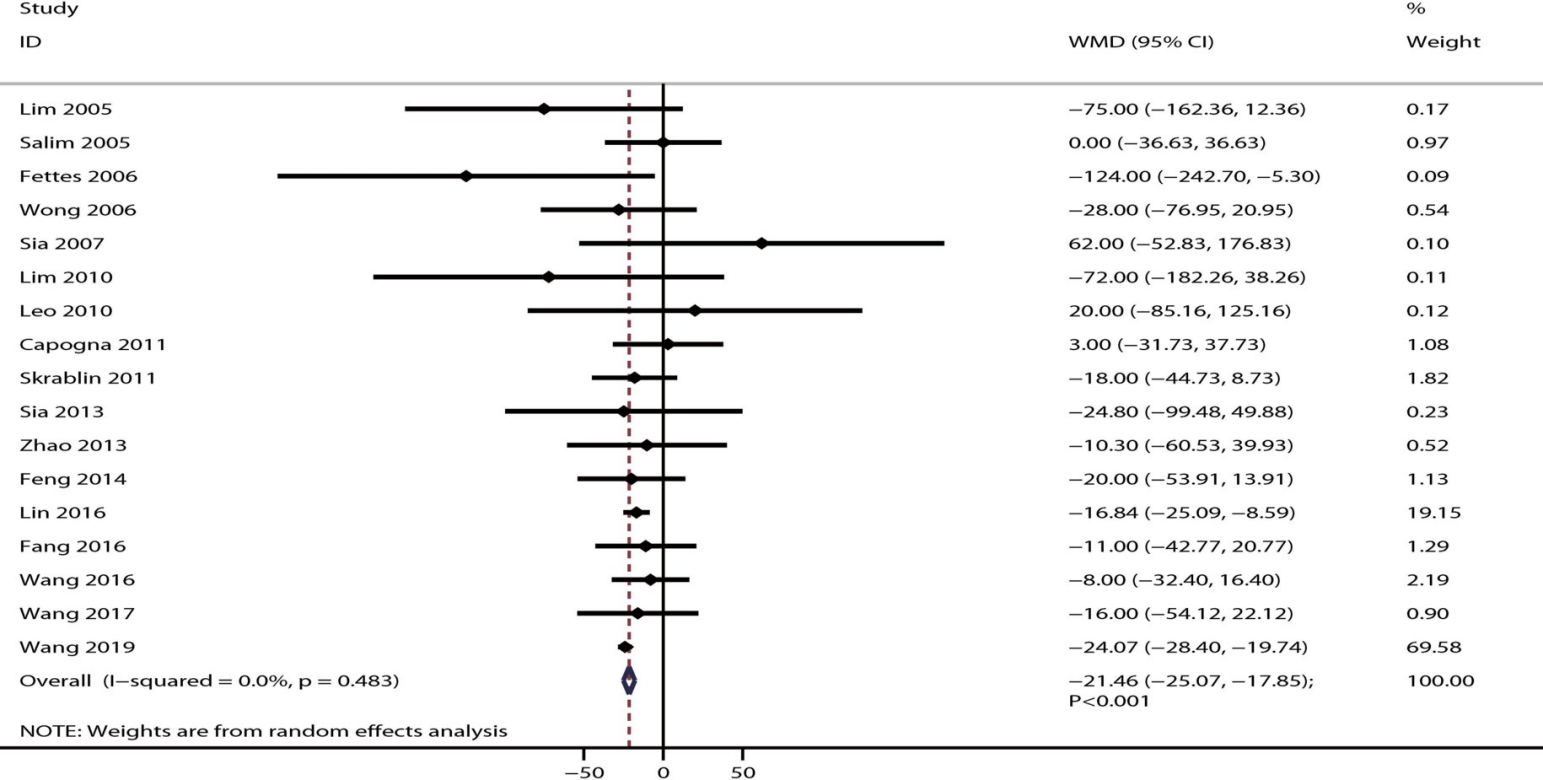
The effect of IPB versus CPI on total duration of labor.

The data for the effect of IPB versus CPI on the total duration of labor were available in 17 studies. It was noted that IPB was associated with a shorter total duration of labor [WMD: −21.46; 95% CI: −25.07 to −17.85; P < 0.001; no evidence of heterogeneity (*I*^*2*^ = 0.0%; P = 0.483); as shown in Fig 4]. Sensitivity analysis indicated that the conclusion was stable and not changed by excluding any particular trial (S3 Appendix). Moreover, this significant difference remained irrespective of PCEA status (Table 2). The symmetry for funnel plot indicated potential publication bias, but the Egger (P = 0.334) and Begg (P = 0.343) tests suggested no significant publication bias (S4 Appendix).

**Fig 5:**
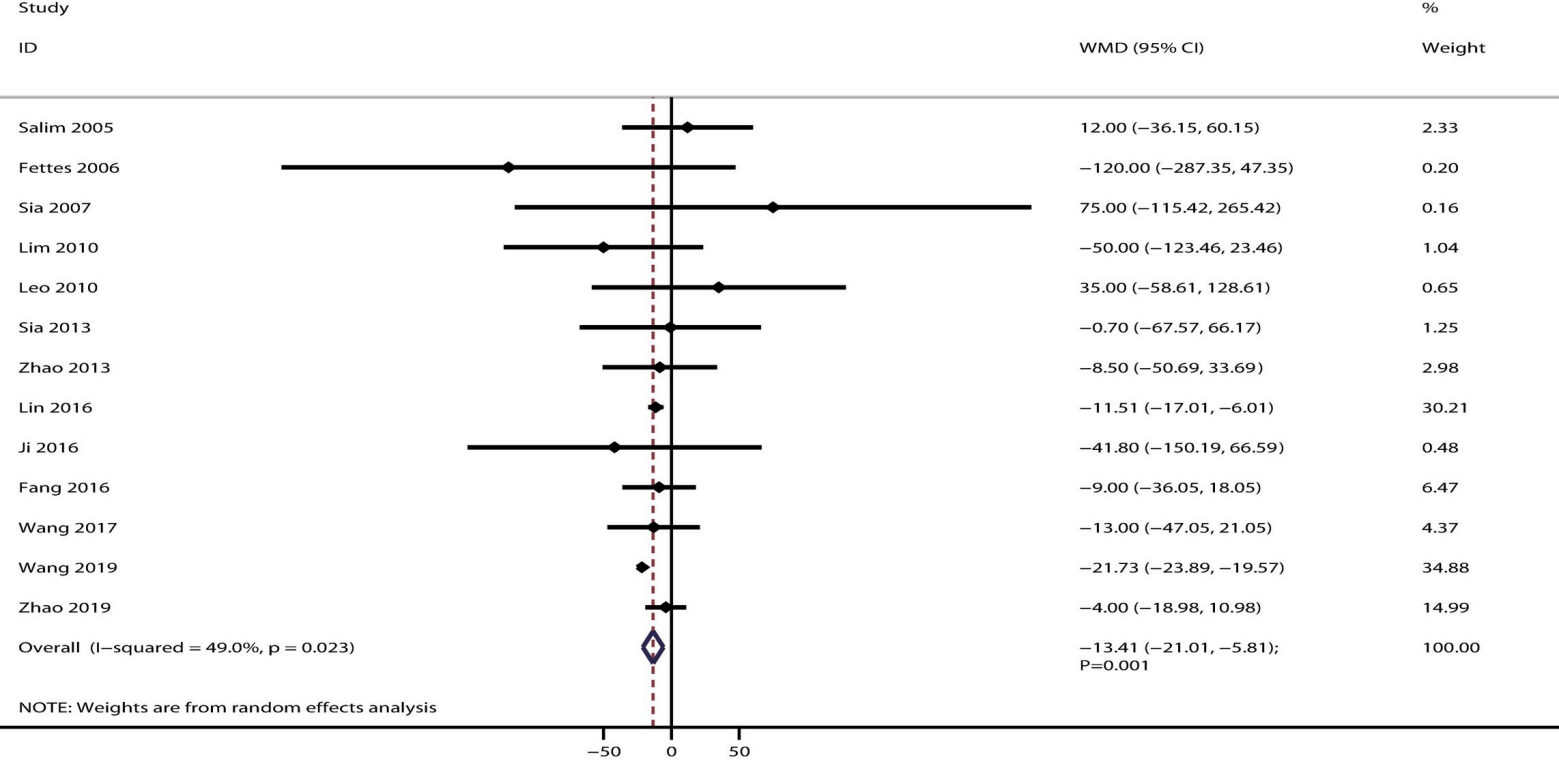
The effect of IPB versus CPI on the duration of the first stage of labor.

The data for the effect of IPB versus CPI on the duration of the first stage of labor were available in 13 studies. The duration of the first stage of labor in parturients treated with IPB was significantly shorter than that of those treated with CPI [WMD: −13.41; 95% CI: −21.01 to −5.81; P = 0.001; potential significant heterogeneity (*I*^*2*^ = 49.0%; P = 0.023); Fig 5]. The conclusion was stable and not sequentially altered by excluding any individual trial (S3 Appendix). Subgroups analysis indicated that this significant difference was mainly detected in parturients who received PCEA (Table 2). Although the symmetry for funnel plot could not rule out publication bias, there was no significant publication bias for the duration of the first stage of labor (P-values for Egger and Begg tests: 0.131 and 0.360, respectively; S4 Appendix).

**Fig 6:**
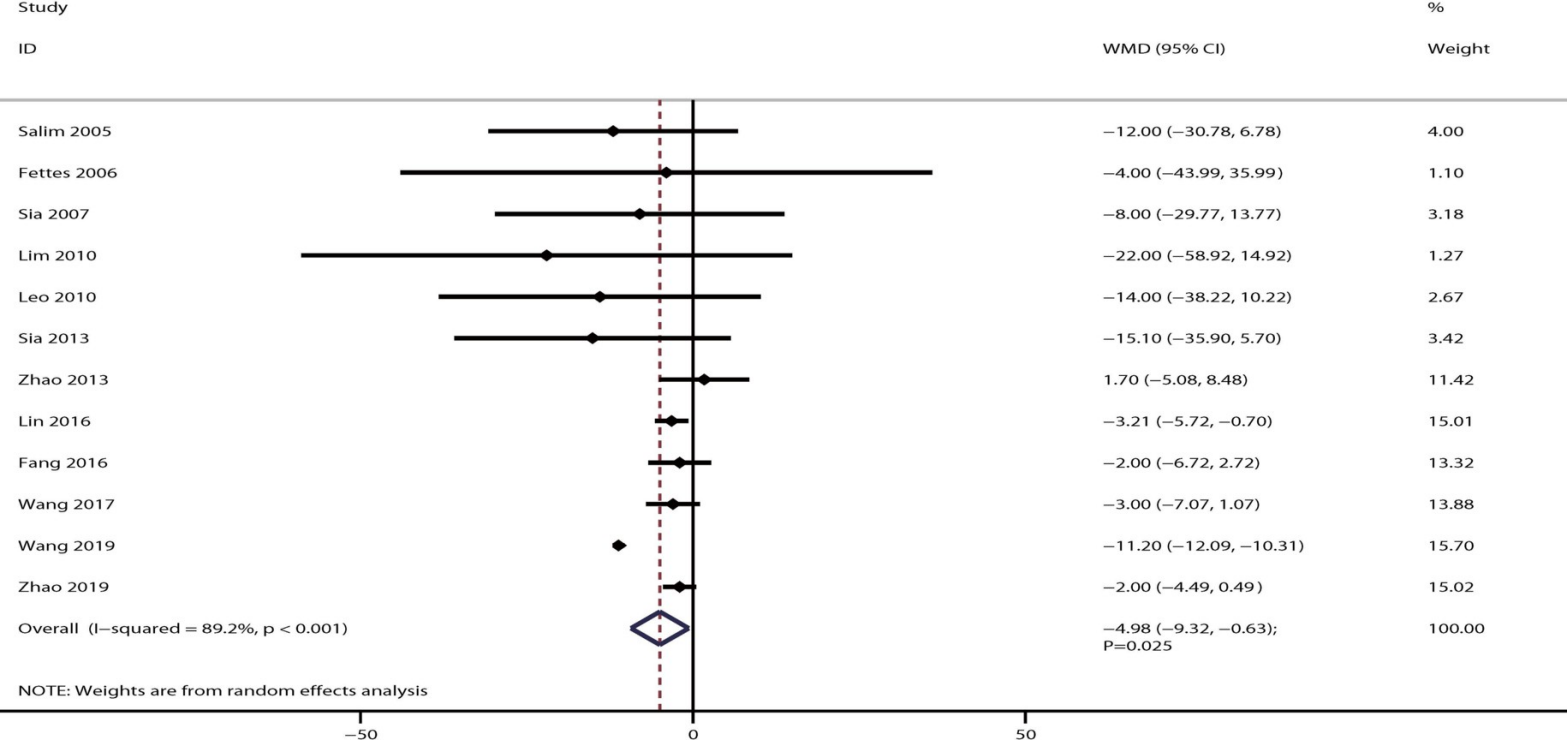
The effect of IPB versus CPI on the duration of the second stage of labor.

The data for the effect of IPB versus CPI on the duration of the second stage of labor were available in 12 studies. The summary of WMD indicated that IPB was associated with a shorter duration of the second stage of labor as compared with CPI [WMD: −4.98; 95% CI: −9.32 to −0.63; P = 0.025; significant heterogeneity (*I*^*2*^ = 89.2%; P < 0.001); Fig 6]. The pooled conclusion was not sequentially altered by excluding any individual trial (S3 Appendix). Subgroup analysis indicated that there was a difference between IPB and CPI on the duration of the second stage of labor when parturients received PCEA (Table 2). The symmetry for funnel plot was relative good, and no significant publication bias was observed by the Egger (P = 0.160) and Begg (P = 0.150) tests (S4 Appendix).

### Anesthetic interventions

**Fig 7:**
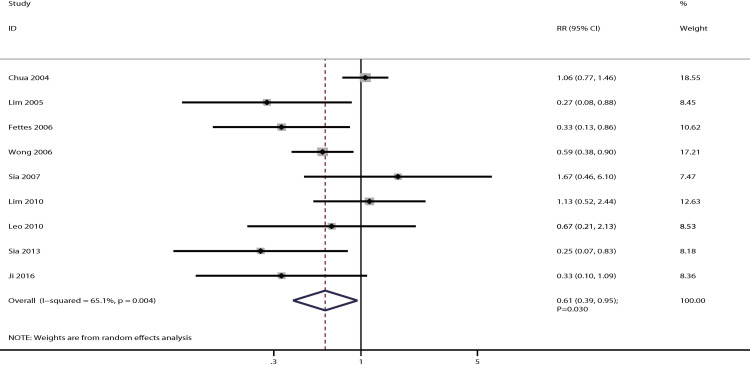
The effect of IPB versus CPI on the incidence of required anesthetic interventions.

The data for the effect of IPB versus CPI on the incidence of required anesthetic interventions were available in nine studies. Parturients treated with IPB were associated with a lower incidence of required anesthetic interventions than those treated with CPI [RR: 0.61; 95% CI: 0.39–0.95; P = 0.030; significant heterogeneity (*I*^*2*^ = 65.1%; P = 0.004); Fig 7]. The conclusion was variable owing to marginal 95% CI through sensitivity analysis (S3 Appendix). Subgroup analysis indicated that there was a significant difference between IPB and CPI on required anesthetic interventions mainly in parturients who received PCEA (Table 2). Review of the funnel plot could not rule out potential publication bias; however, the Egger (P = 0.127) and Begg (P = 0.348) test results suggested no significant publication bias (S4 Appendix).

**Fig 8:**
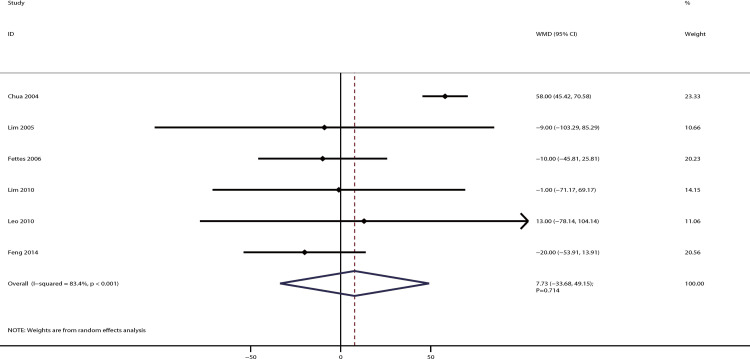
The effect of IPB versus CPI on the time to the first required anesthetic intervention.

The data for the effect of IPB versus CPI on the time required for first anesthetic intervention were available in six studies. There was no significant difference between IPB and CPI on the time required for first anesthetic intervention [WMD: 7.73; 95% CI: −33.68 to 49.15; P = 0.714; significant heterogeneity (*I*^*2*^ = 83.4%; P < 0.001); Fig 8)]. This non-significant difference between IPB and CPI remained and was not sequentially altered by excluding any individual trial (S3 Appendix). The results of subgroup analysis were consistent with overall analysis irrespective of PCEA status (Table 2). The symmetry for funnel plot was general, and quantitative analysis found no significant publication bias for the time required for the first anesthetic intervention (P-values for Egger and Begg tests: 0.091 and 0.707, respectively; S4 Appendix).

### Milligrams per hour of local anesthetic (bupivacaine equivalents)

**Fig 9:**
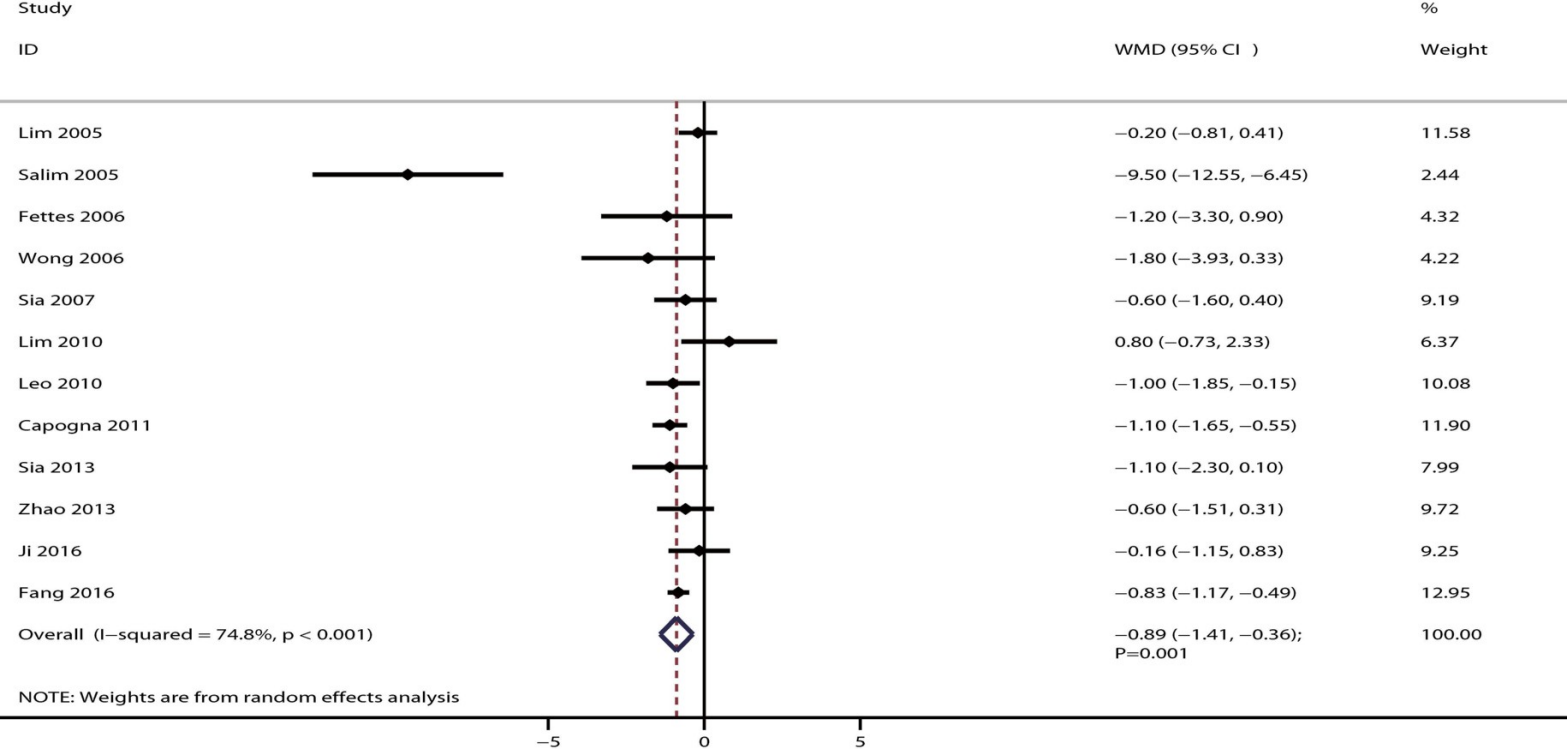
The effect of IPB versus CPI on the milligrams per hour of local anesthetic (bupivacaine equivalents).

The data for the effect of IPB versus CPI on local anesthetic (bupivacaine equivalents) in milligrams per hour were available in 12 studies. It was noted that IPB was associated with local anesthetic (bupivacaine equivalents) in lower milligrams per hour compared with CPI [WMD: −0.89; 95% CI: −1.41 to −0.36; P = 0.001; significant heterogeneity (*I*^*2*^ = 74.8%; P < 0.001); Fig 9]. The conclusion was stable after sequentially excluding any individual trial (S3 Appendix). This significant difference was mainly observed for parturients who received PCEA (Table 2). The symmetry for funnel plot was not good, while publication bias was not associated with statistical significance (P-values for Egger and Begg tests: 0.355 and 0.373, respectively; S4 Appendix).

### Maternal satisfaction

**Fig 10:**
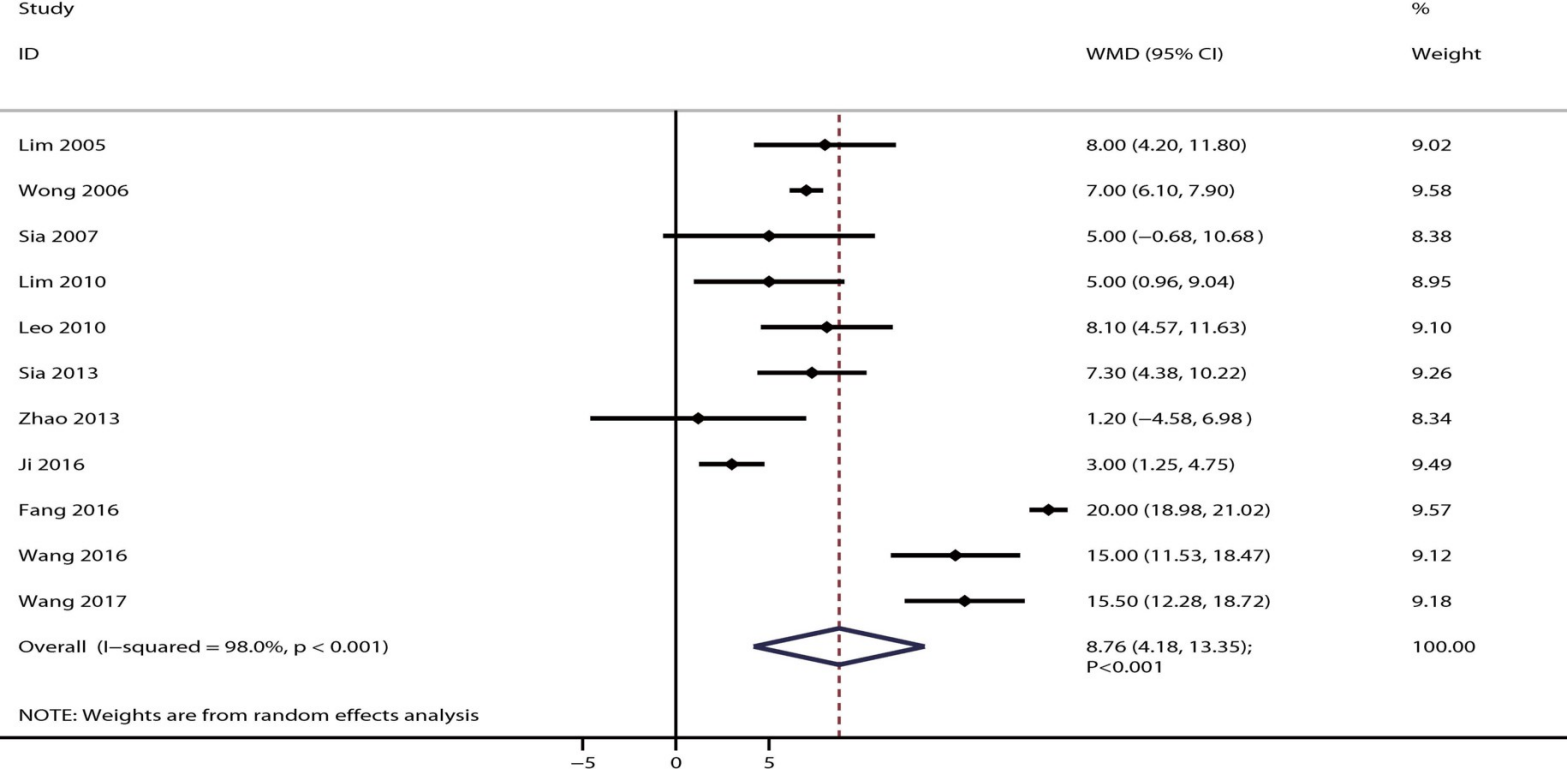
The effect of IPB versus CPI on maternal satisfaction.

The data for the effect of IPB versus CPI on maternal satisfaction were available in 11 studies. It was noted that IPB could significantly improve maternal satisfaction compared with CPI [WMD: 8.76; 95% CI: 4.18–13.35; P < 0.001; significant heterogeneity (*I*^*2*^ = 98.0%; P < 0.001); Fig 10]. The pooled conclusion was not altered by excluding any specific trial (S3 Appendix), and this significant improvement remained irrespective of PECA status (Table 2). The summary for funnel plot could not rule out potential publication bias, but quantitative analysis found no significant publication bias (P-values for Egger and Begg tests: 0.514 and 0.755, respectively; S4 Appendix).

### Adverse events

The summary results of IPB versus CPI on the risk of specific adverse events are shown in Table 3. There was no significant difference between IPB and CPI on the risk of motor blockade (RR: 0.44; 95% CI: 0.19–1.03; P = 0.058), pruritus (RR: 1.03; 95% CI: 0.87–1.23; P = 0.703), nausea and vomiting (RR: 1.44; 95% CI: 0.57–3.64; P = 0.435), shivering (RR: 0.98; 95% CI: 0.72–1.34; P = 0.913), hypotension (RR: 1.43; 95% CI: 0.95–2.16; P = 0.084), intrapartum fever (RR: 0.63; 95% CI: 0.34–1.18; P = 0.149), perineal tears (RR: 1.44; 95% CI: 0.77–2.70; P = 0.258), retained placental products (RR: 1.00; 95%CI: 0.29–3.50; P = 0.998), postpartum hemorrhage (RR: 0.73; 95% CI: 0.22–2.44; P = 0.607), and fetal bradycardia (RR: 1.04; 95% CI: 0.32–3.40; P = 0.944).

**Table 3:** The summary results for adverse events.

Outcomes	No. of studies	RR and 95% CI	P-value	*I*^*2*^ (%)	P-value for Q-statistic
Motor blockade	10	0.44 (0.19–1.03)	0.058	65.8	0.002
Pruritus	8	1.03 (0.87–1.23)	0.703	0.0	0.958
Nausea and vomiting	7	1.44 (0.57–3.64)	0.435	57.2	0.029
Shivering	5	0.98 (0.72–1.34)	0.913	39.8	0.156
Hypotension	10	1.43 (0.95–2.16)	0.084	0.0	0.756
Intrapartum fever	7	0.63 (0.34–1.18)	0.149	51.2	0.056
Perineal tears	2	1.44 (0.77–2.70)	0.258	0.0	0.586
Retained placental products	2	1.00 (0.29–3.50)	0.998	0.0	0.511
Postpartum hemorrhage	3	0.73 (0.22–2.44)	0.607	9.1	0.333
Fetal bradycardia	3	1.04 (0.32–3.40)	0.944	0.0	0.711

## Discussion

This study was based on published RCTs, and it compared the efficacy and safety of IPB versus CPI for labor analgesia through a meta-analytic approach. The analysis recruited 2,573 parturients from 22 RCTs across various individuals’ characteristics. This study determined that IPB could shorten the total duration of labor and duration of the first and second stage of labor, reduce requirements for additional anesthetic intervention, and improve maternal satisfaction. However, there was no significant difference between IPB and CPI for the mode of delivery and specific adverse events. Furthermore, the treatment effects of IPB versus CPI on required anesthetic interventions, the time required for first anesthetic intervention, local anesthetic (bupivacaine equivalents) in milligrams per hour, and maternal satisfaction could be affected by PECA status.

Several systematic reviews and meta-analyses have addressed the treatment efficacy of IPB versus CPI for labor analgesia [43, 44]. George et al. [43] conducted a meta-analysis of nine studies and observed that IEB was associated with slightly reduced local anesthetic usage and improved maternal satisfaction as compared with CPI. Moreover, a meta-analysis conducted by Xu et al. [44] included 11 studies and observed that IEB with PCEA significantly reduced the incidences of instrumental delivery, breakthrough pain, PCEA usage rates, and the use of local anesthetics. In addition, it was pointed out that labor duration was significantly shorter and that maternal satisfaction was significantly improved in those who received IEB with PCEA compared with those who received CPI with PCEA. However, several important studies were not included, and the results need to be updated. Therefore, the current meta-analysis was conducted based on RCTs to determine the efficacy and safety of IPB versus CPI for labor analgesia.

The summary of the results suggested that the mode of delivery was not affected by IEB or CPI because studies reported no significant difference between IEB and CPI for the incidences of cesarean delivery and instrumental delivery. The study conducted by Skrablin et al. [30] reported that IEB was associated with a lower incidence of cesarean delivery as compared with CPI. Moreover, two included studies revealed that the incidence of instrumental delivery in the IEB group was significantly lower than that in the CPI group [29, 40]. However, the study conducted by Nunes et al. [35] showed that IEB was associated with a high incidence of instrumental delivery. It was pointed out that extensive blocking of IEB causes relaxation of the soft birth canal, softening of the cervix, and a smooth descent of the soft fetal head, suggesting that IEB pulse injection did not affect contractile reduction of the pelvic floor muscle tone [35].

The present study observed that the duration of labor was significantly reduced in the IEB group, irrespective of the total duration of labor and the duration of the first and second stage of labor. The potential reason for this could be that the analgesic effect of IEB was better than that of CPI, possibly reducing the risk of maternal motor block and promoting the progression of labor. Moreover, the incidence required for additional anesthetic interventions was significantly lower in the IEB group than in the CPI group, although no significant difference were observed between the groups for the time required for the first anesthetic intervention. The potential reason for this could be the uniform distribution of the drug in the IEB group in the epidural space, close contact with the spinal nerve root, and more extensive blockade [36]. Furthermore, maternal satisfaction in the IEB group was significantly improved compared with the CPI group. The potential reason for this significantly correlated with the requirement for additional anesthetic interventions, which was mostly used for suppressing a transitory exacerbation of pain [45, 46]. Finally, the incidence of adverse events between IEB and CPI were not statistically significant, which may have been due to lower event rates and insufficient power for the detection of potential difference between IEB and CPI.

This study has several limitations. First, the heterogeneity of the included trials was not fully interpreted via the use of sensitivity and subgroups analyses. Second, the strategies of analgesia differed across the included studies, possibly affecting the results of this study. Third, the details regarding mechanical delivery interventions were not reported in most of the included studies, affecting the safety and treatment efficacy of IPB with CPI for labor analgesia. Forth, individual data were not available, restricting a more detailed analysis. Finally, the analysis based on published articles, the inclusion of studies published in English and Chinese, and publication bias were inevitable problems.

### Conclusion

The findings of this study revealed that the models of delivery were not different between IEB and CPI. Moreover, the duration of labor, irrespective of the total stages, first stage, and second stage was significantly shorter in the IEB group than in the CPI group. Furthermore, the requirements for additional anesthetic interventions were significantly reduced and maternal satisfaction was significantly improved in the IEB group. Further large-scale RCTs should be conducted to verify the findings of this study to assess differences according to parity status.

## Supporting information

S1 Appendix Search strategy in PubMed. (DOCX)

S2 Appendix Abstracted data. (XLSX)

S3 Appendix Figures of sensitivity. (DOCX)

S4 Appendix Figures for the funnel plot. (DOCX)

S1 Checklist PRISMA 2009 checklist. (DOC)
