# Supplementary material for: Intermittent epidural bolus versus continuous epidural infusions for labor analgesia: A meta-analysis of randomized controlled trials
Source: PLoS One. 2020 Jun 12;15(6):e0234353. doi: 10.1371/journal.pone.0234353 (PMC7292420; doi:10.1371/journal.pone.0234353)
Supplement: S1 Appendix — (DOCX) [file pone.0234353.s001.docx]

**Search strategy in PubMed:**

((((Pregnancy) AND epidural analgesia) AND (((programmed) OR automated) OR intermittent))) OR (((((((Pregnancies[Title/Abstract]) OR Gestation[Title/Abstract])) OR "Pregnancy"[Mesh])) AND ((Epidural Analgesia[Title/Abstract]) OR "Analgesia, Epidural"[Mesh])) AND (((intermittent [Text Word]) OR automated[Text Word]) OR programmed[Text Word])) AND (Randomized controlled trials)
