# Supplementary material for: Intermittent epidural bolus versus continuous epidural infusions for labor analgesia: A meta-analysis of randomized controlled trials
Source: PLoS One. 2020 Jun 12;15(6):e0234353. doi: 10.1371/journal.pone.0234353 (PMC7292420; doi:10.1371/journal.pone.0234353)
Supplement: S3 Appendix — (DOCX) [file pone.0234353.s003.docx]

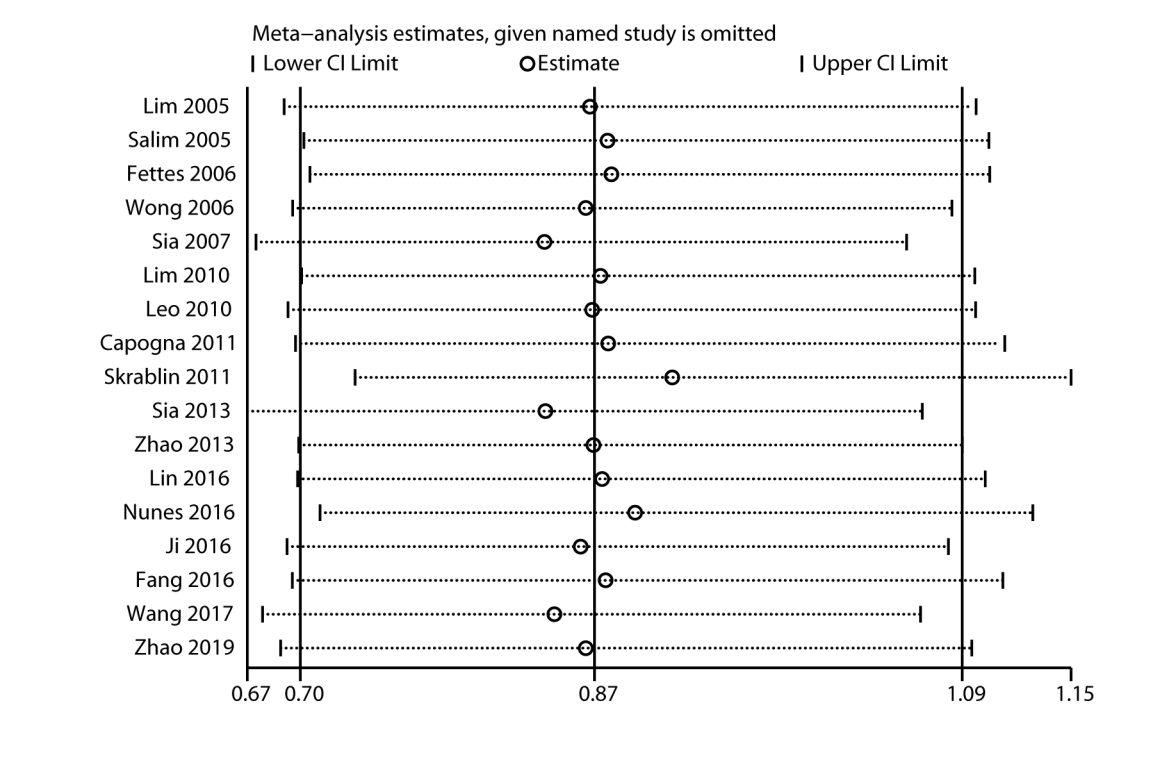


Figure S1. Sensitivity for cesarean delivery


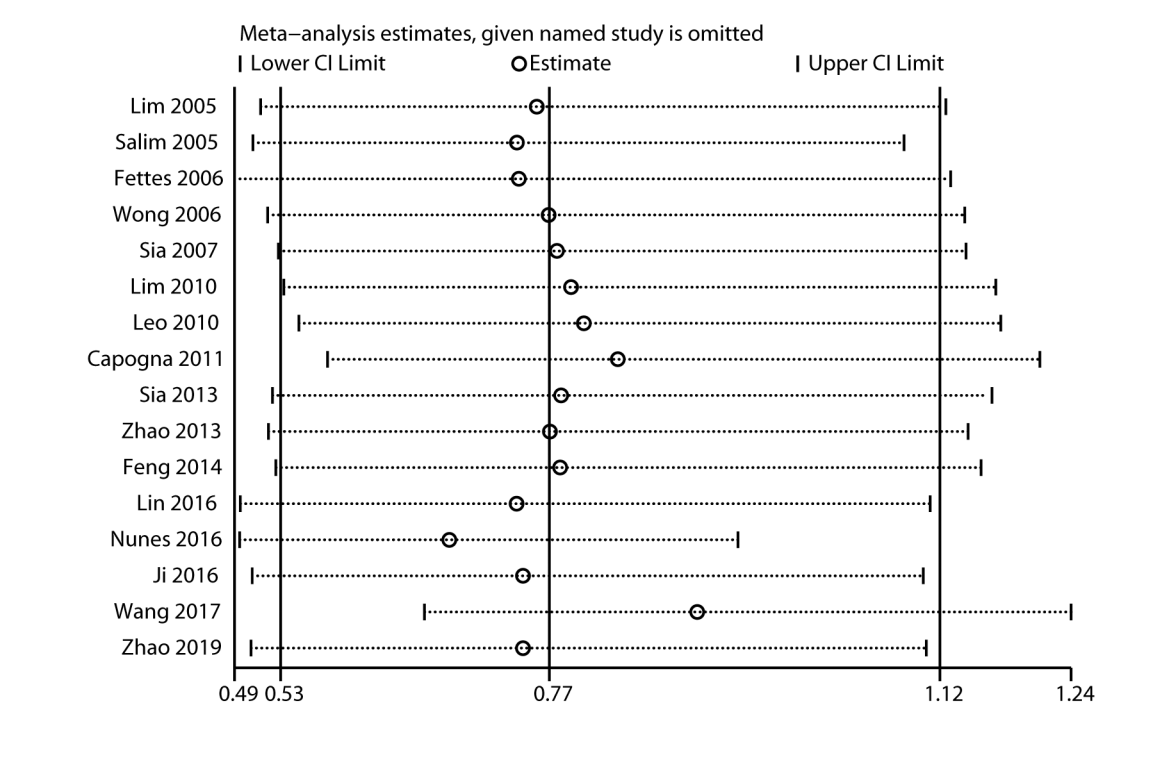


Figure S2. Sensitivity for instrumental delivery


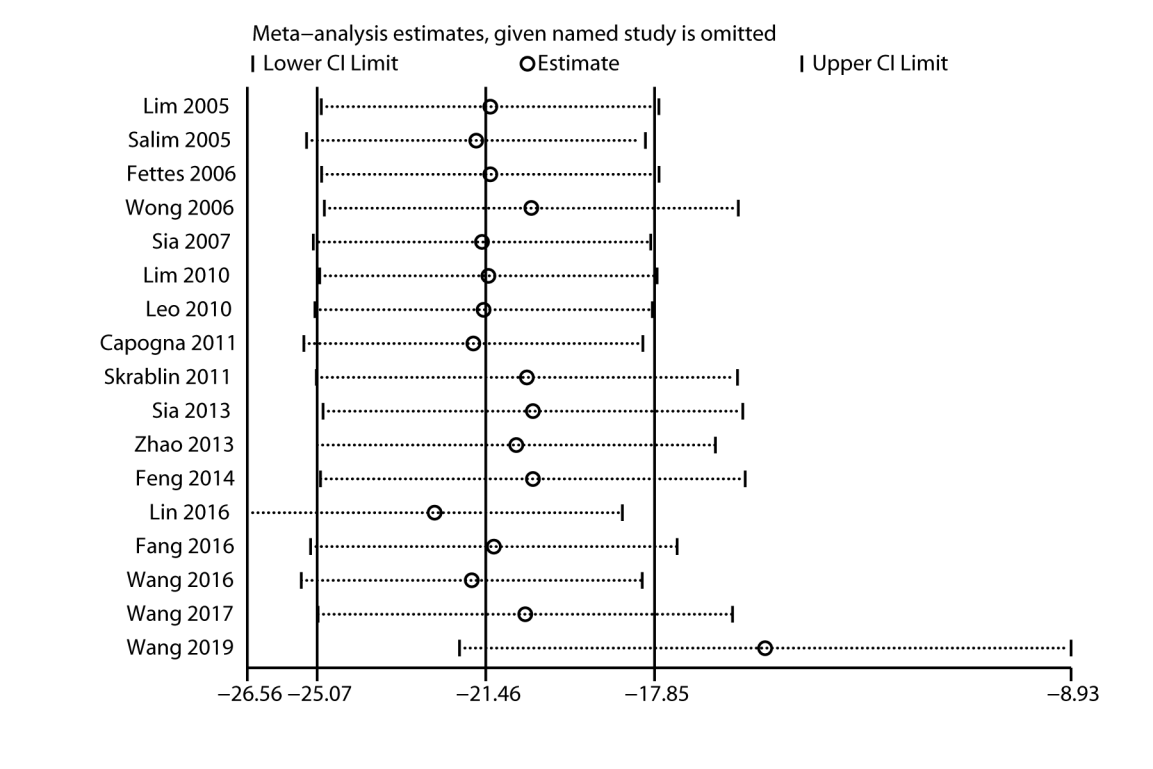


Figure S3. Sensitivity for total duration of labor


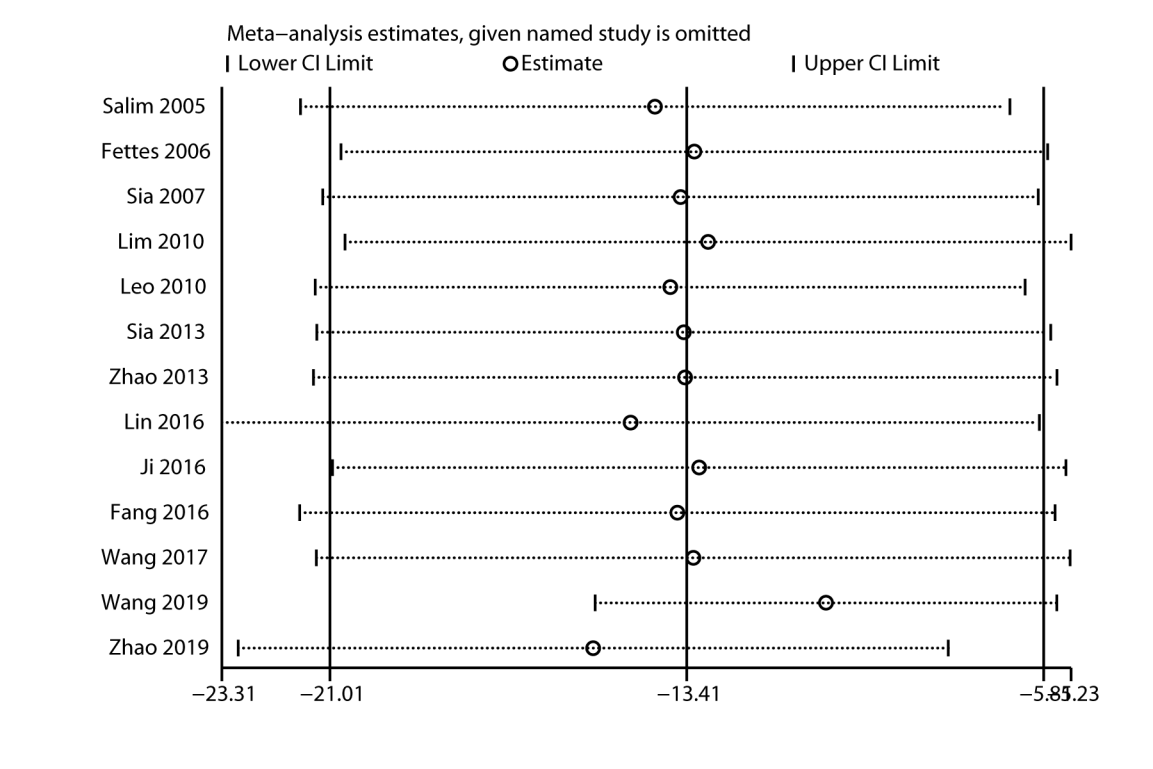


Figure S4. Sensitivity for the duration of first stage of labor


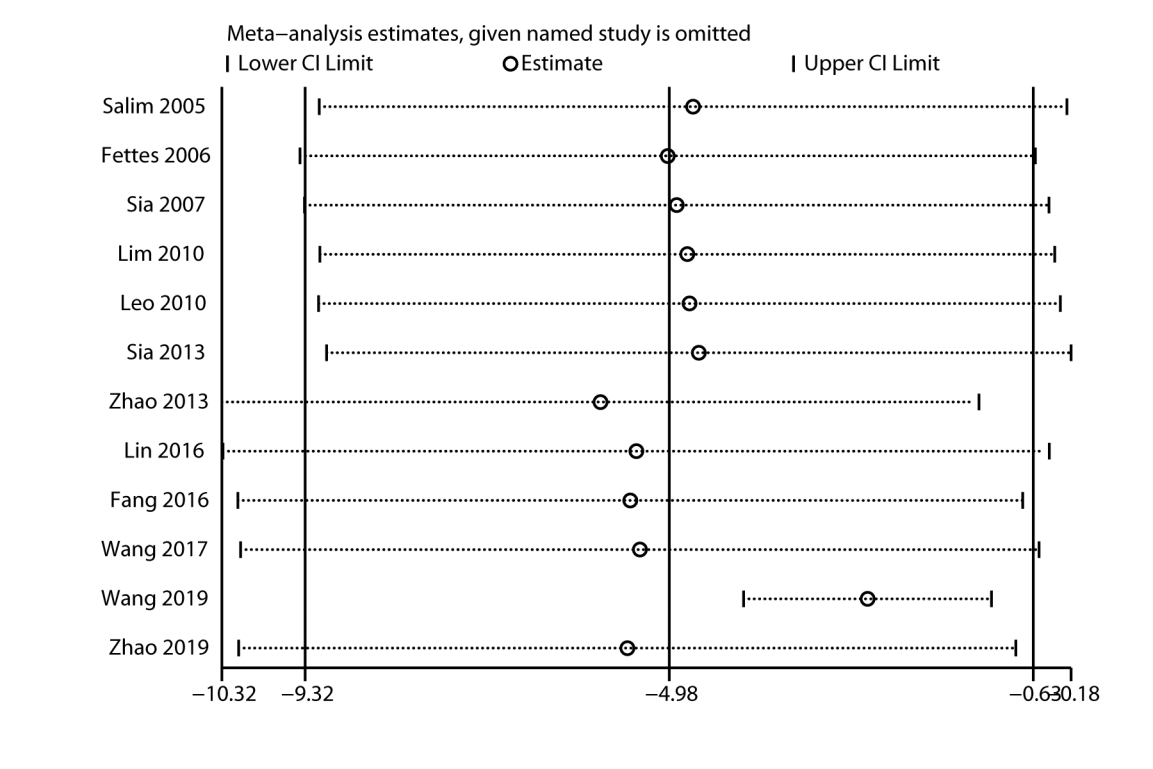


Figure S5. Sensitivity for the duration of second stage of labor


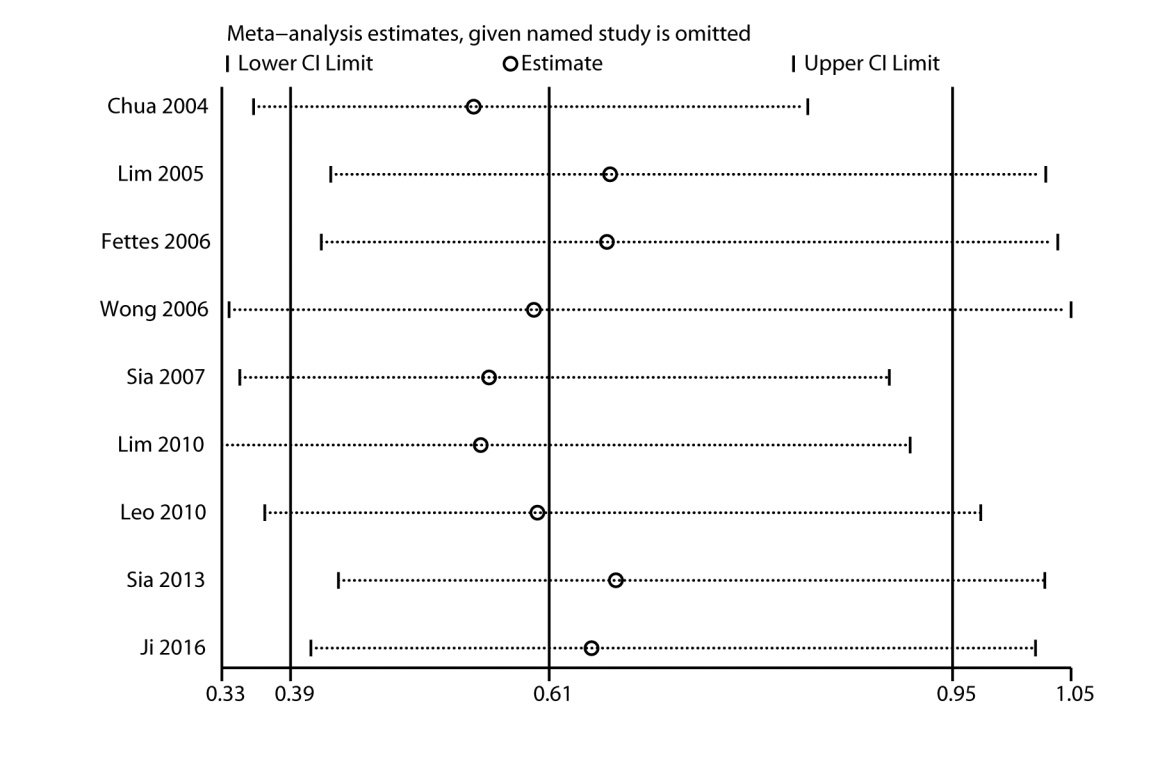


Figure S6. Sensitivity for the incidence of required anesthetic interventions


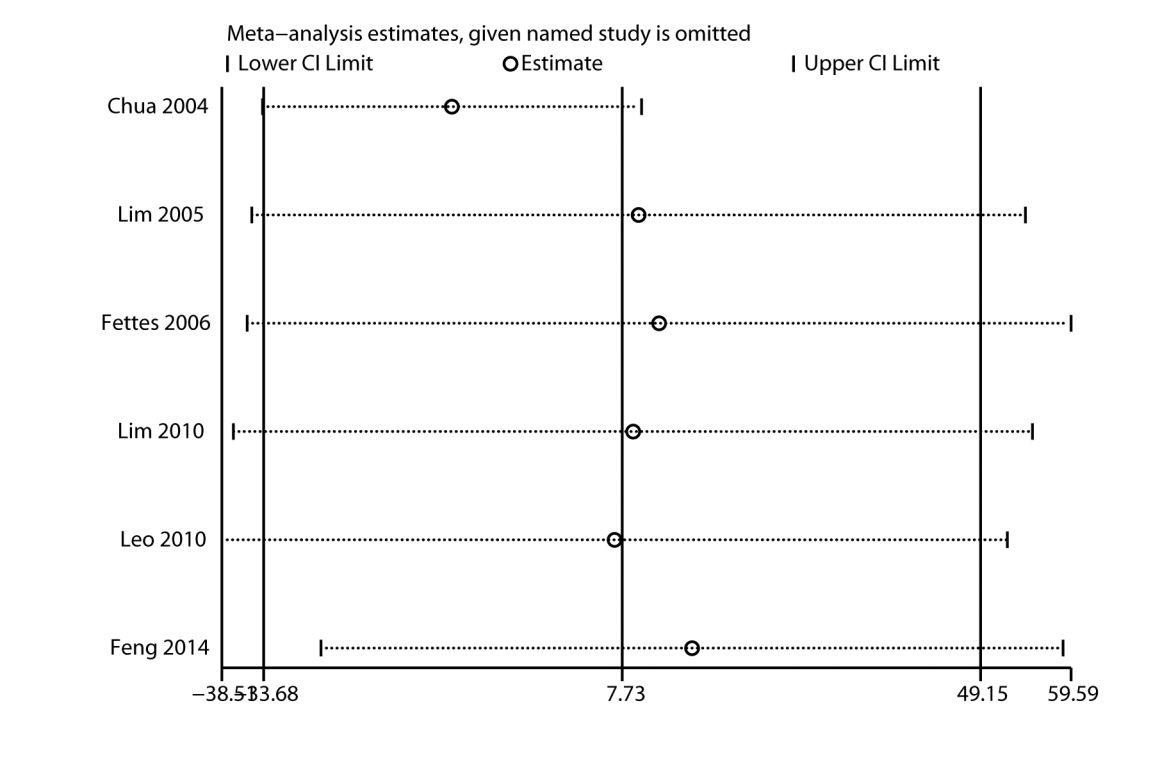


Figure S7. Sensitivity for the time to first required anesthetic intervention


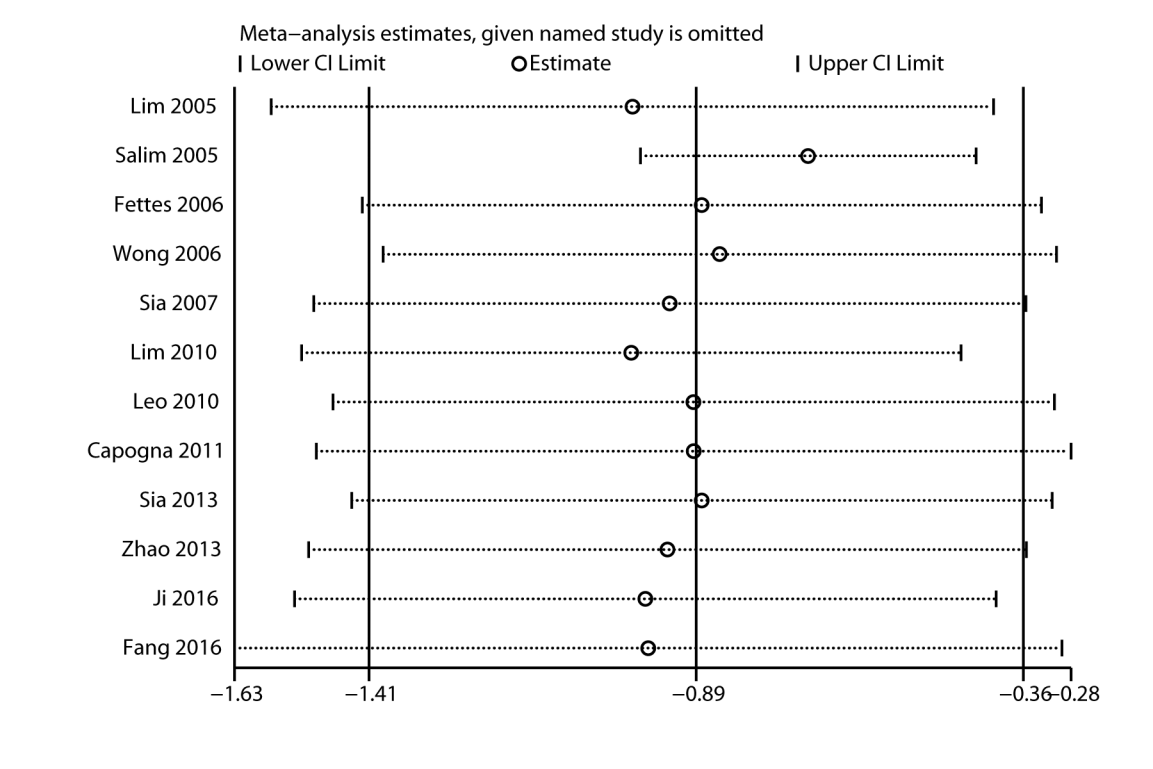


Figure S8. Sensitivity for the milligrams per hour of local anesthetic (bupivacaine equivalents)


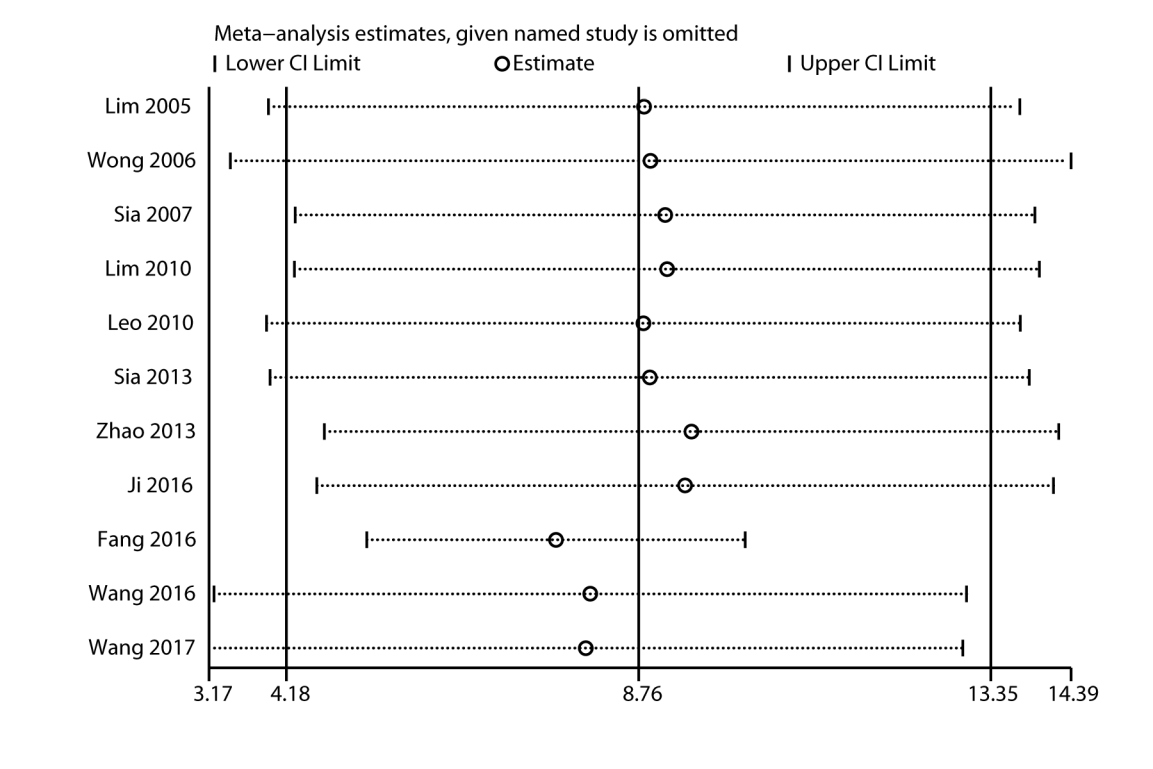


Figure S9. Sensitivity for maternal satisfaction
