# Supplementary material for: Intermittent epidural bolus versus continuous epidural infusions for labor analgesia: A meta-analysis of randomized controlled trials
Source: PLoS One. 2020 Jun 12;15(6):e0234353. doi: 10.1371/journal.pone.0234353 (PMC7292420; doi:10.1371/journal.pone.0234353)
Supplement: S4 Appendix — (DOCX) [file pone.0234353.s004.docx]

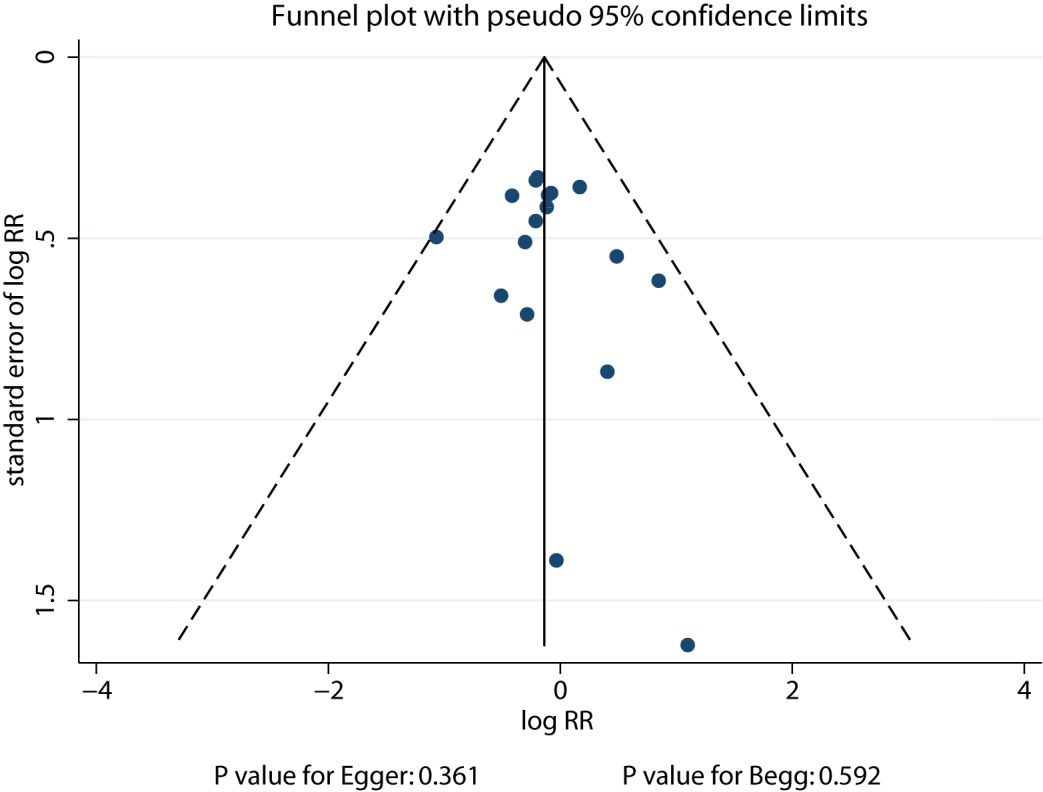


Figure S1. Funnel plot for cesarean delivery


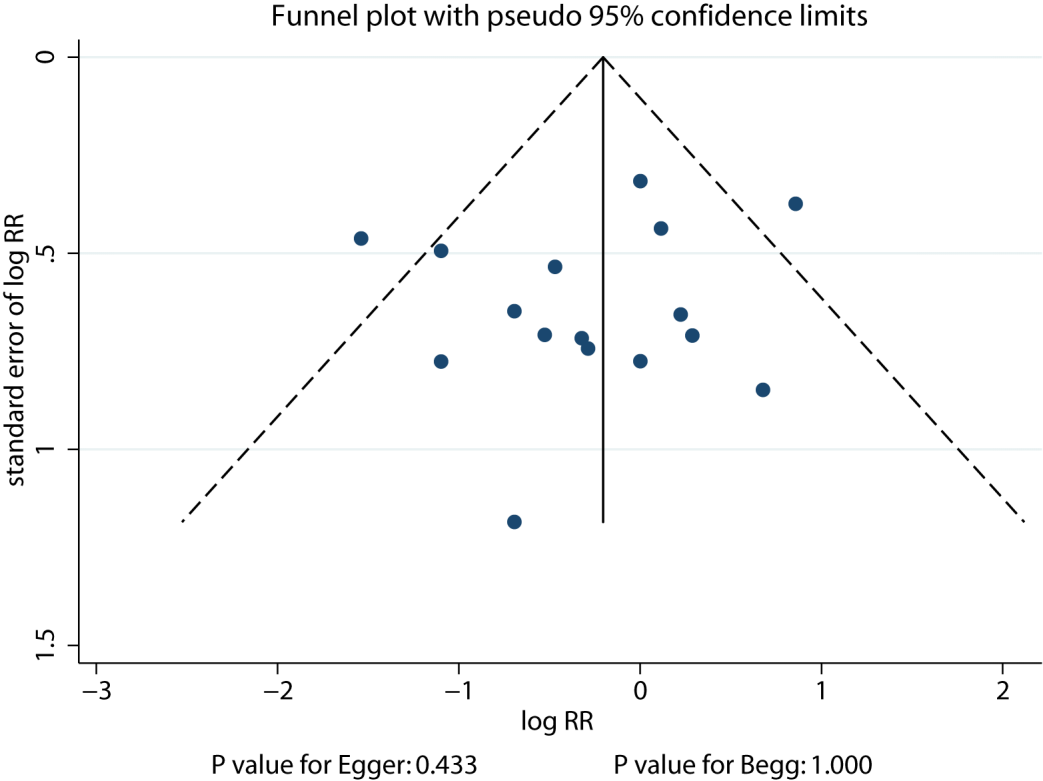


Figure S2. Funnel plot for instrumental delivery


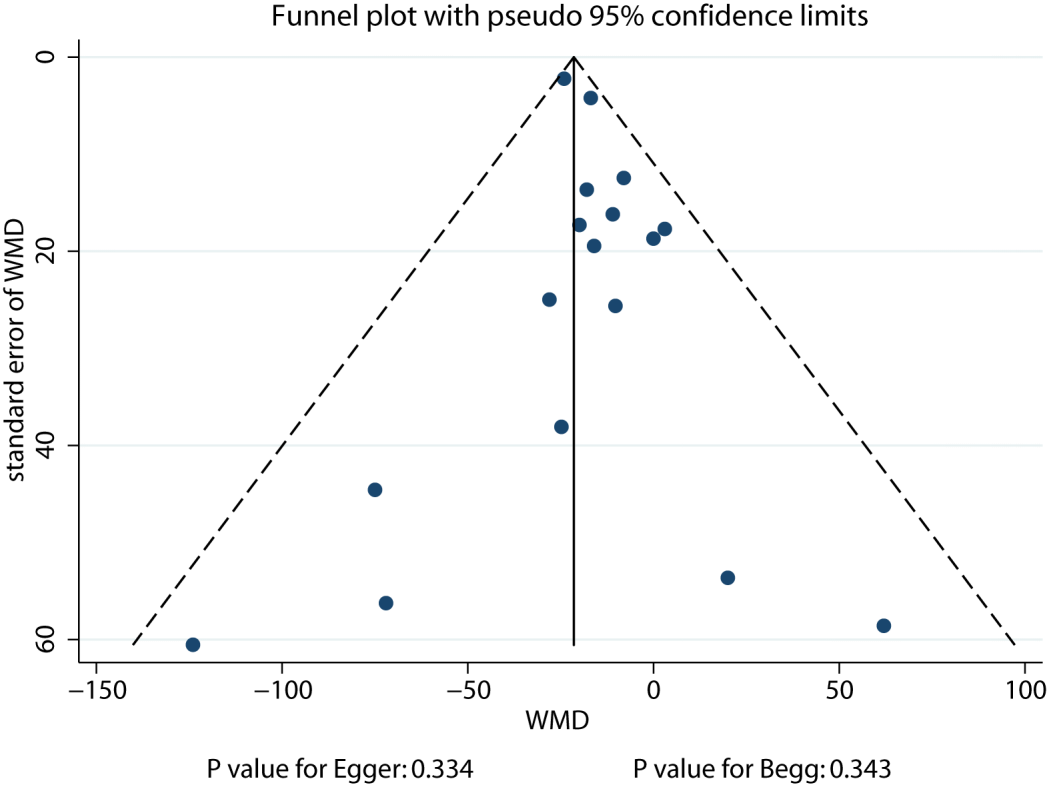


Figure S3. Funnel plot for total duration of labor


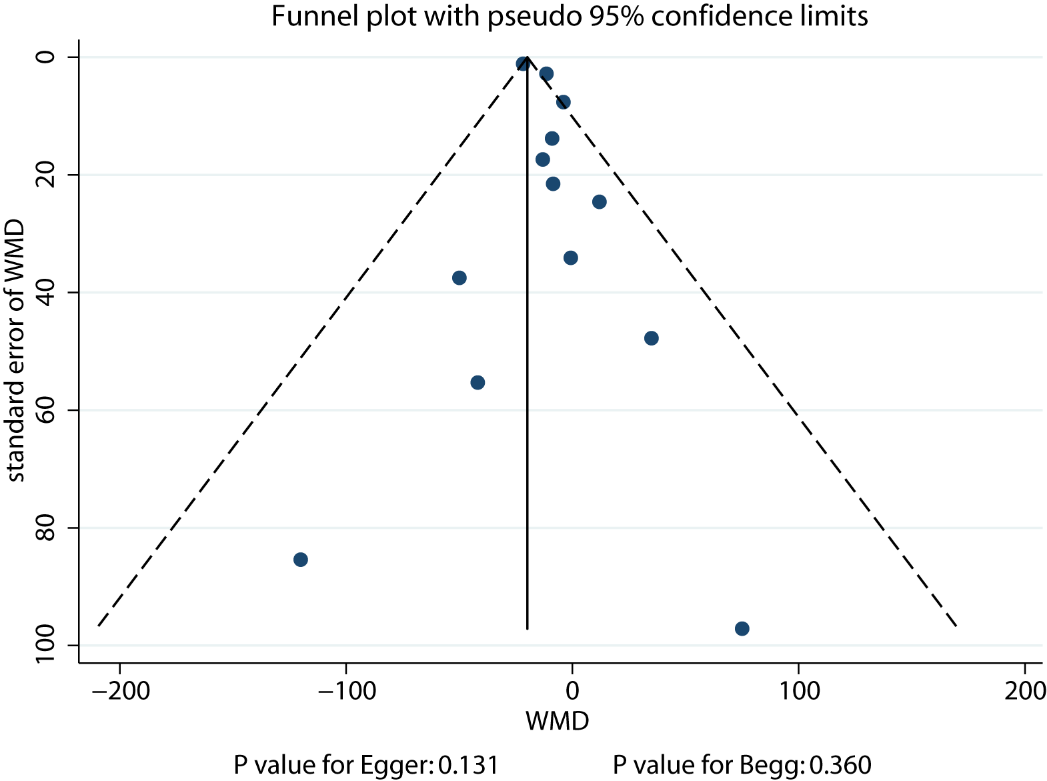


Figure S4. Funnel plot for the duration of first stage of labor


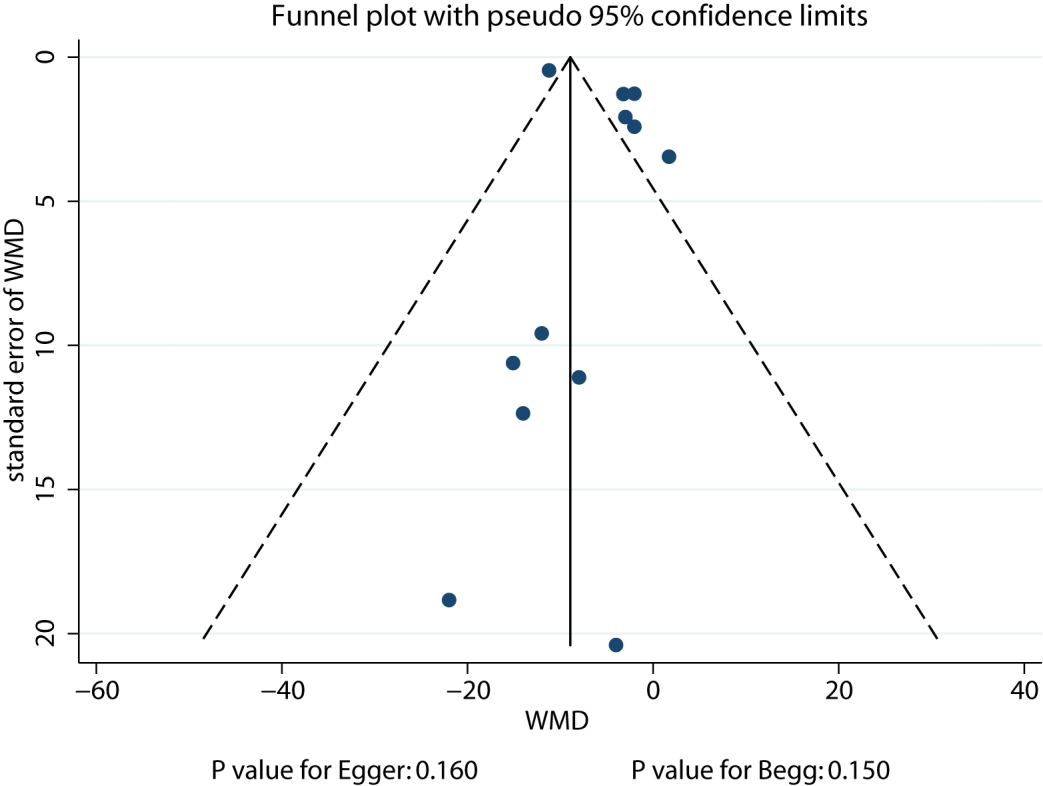


Figure S5. Funnel plot for the duration of second stage of labor


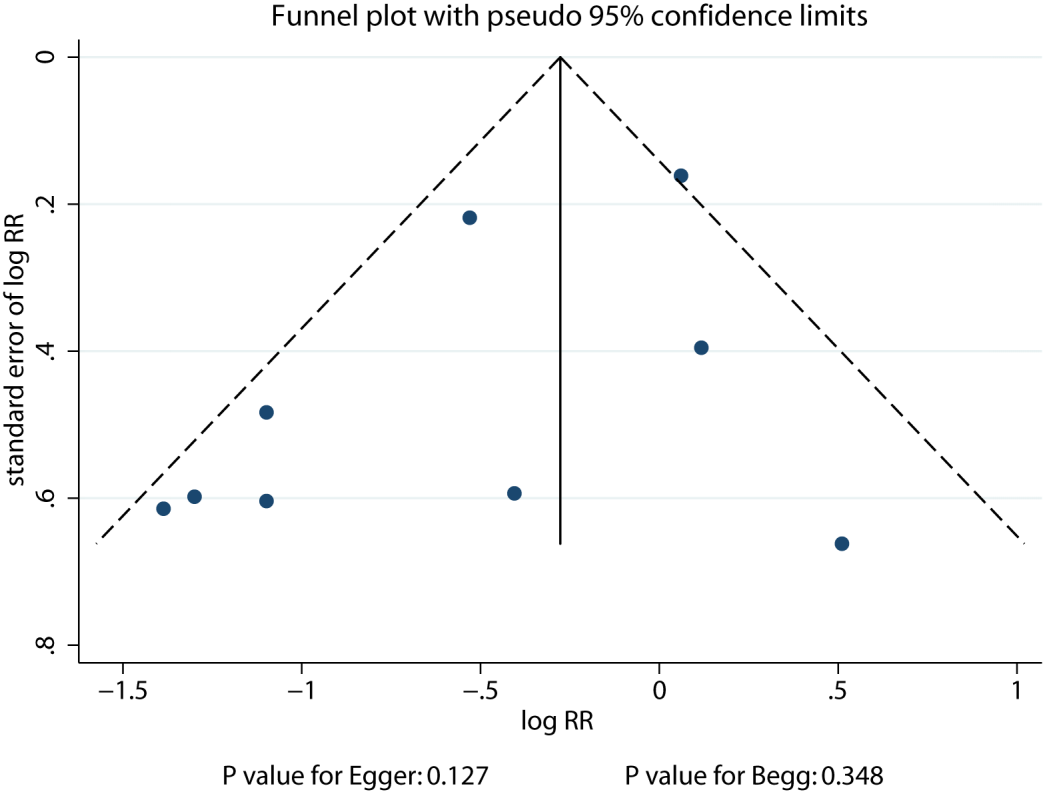


Figure S6. Funnel plot for the incidence of required anesthetic interventions


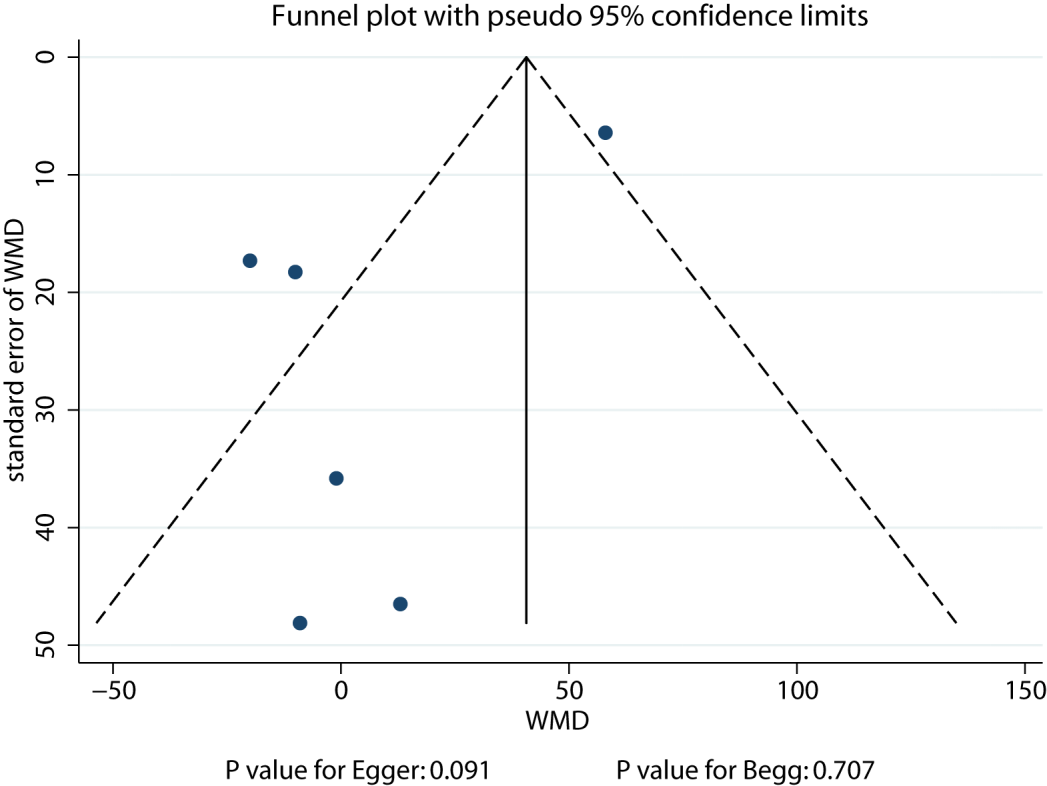


Figure S7. Funnel plot for the time to first required anesthetic intervention


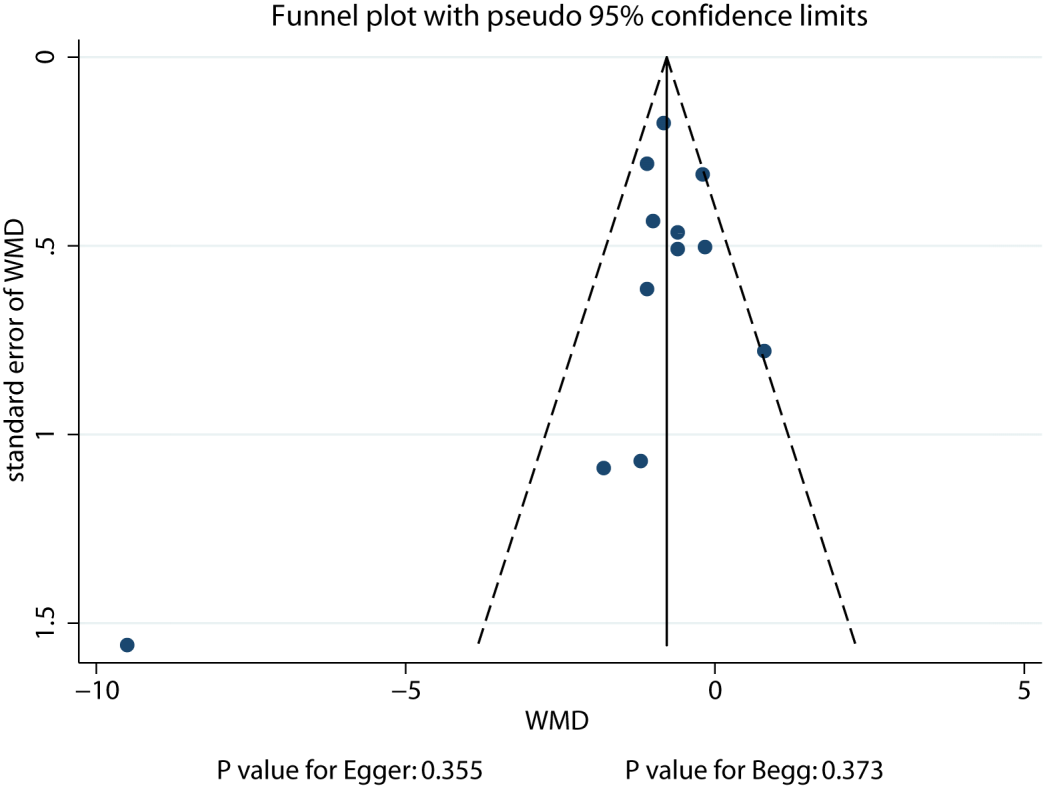


Figure S8. Funnel plot for the milligrams per hour of local anesthetic (bupivacaine equivalents)


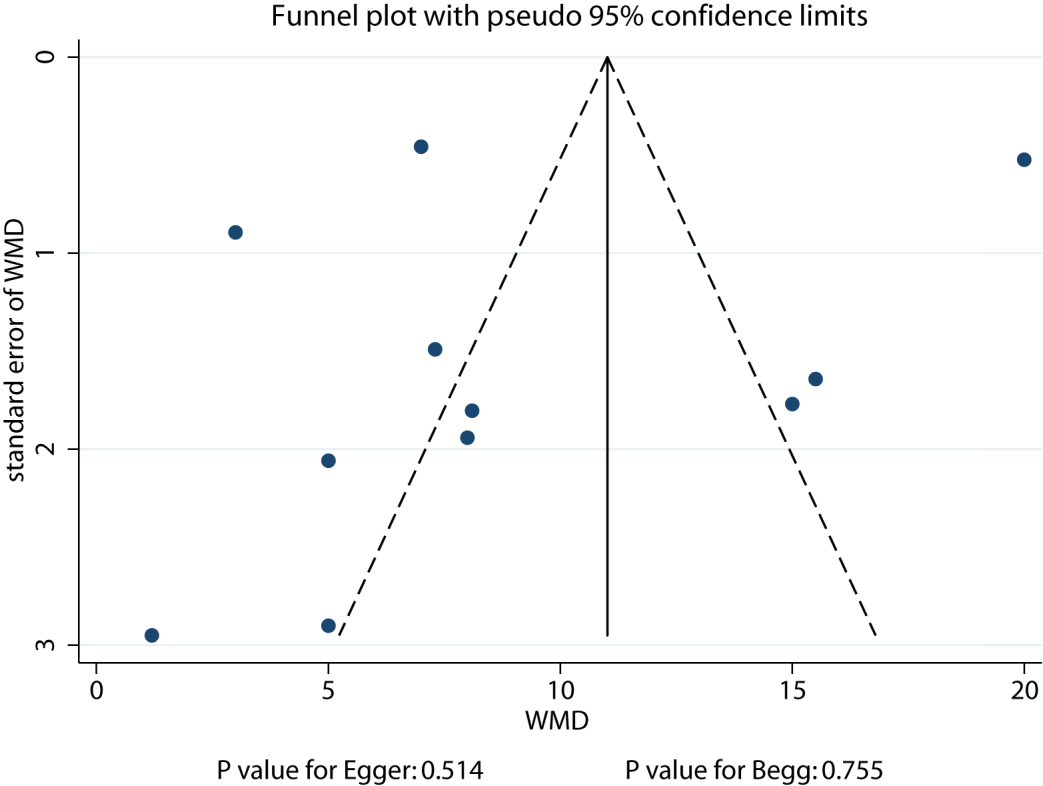


Figure S9. Funnel plot for maternal satisfaction
